# Changes in Physical Activity Patterns Due to the Covid-19 Pandemic: A Systematic Review and Meta-Analysis

**DOI:** 10.3390/ijerph19042250

**Published:** 2022-02-16

**Authors:** Kathrin Wunsch, Korbinian Kienberger, Claudia Niessner

**Affiliations:** Institute of Sports and Sports Science, Karlsruhe Institute of Technology, 76131 Karlsruhe, Germany; korbinian.kienberger@student.kit.edu (K.K.); claudia.niessner@kit.edu (C.N.)

**Keywords:** physical activity, exercise, training, coronavirus, Covid-19, SARS-CoV-2

## Abstract

With the outbreak of the Corona Virus Disease 19 (Covid-19) in late 2019, governments increasingly imposed containment strategies, including social distancing as well as restricted population movement, potentially having negative impacts on mental and physical health. A growing number of studies have examined the impact of the pandemic on different facets of physical activity (PA); an overview combining these (mixed) results, however, is missing. Thus, the objective of this systematic review and meta-analysis was to investigate whether and to which extent PA changed from before to during the Covid-19 pandemic, taking age, gender, and measurement method into account. The literature search was conducted using PubMed, Web of Science, and Scopus. Results of the main characteristics were descriptively synthesized and analyzed in a meta-analysis quantifying effects of the pandemic on PA divided by age groups, with additional subgroup analyses of the characteristics age, gender, and measurement method being narratively synthesized. Overall, 57 studies with a total sample size of 119,094 participants (N between 10 and 60,560 subjects) from 14 countries worldwide with participants aged between four and 93 years were included. Thirty-two studies revealed a significant decline in PA, whereas only five studies found a significant increase in PA during the Covid-19 pandemic. Fourteen studies revealed mixed results. PA decreased in all age groups, independent of gender. Most self-reported and all device-based measurement methods showed a reduction in PA. However, effects were not found to be significant in all age groups. Nevertheless, the declining trend should be noted and governments should strive to enable PA within periods of pandemic restrictions, or promote alternatives such as digital training to avoid negative health consequences within the population.

## 1. Introduction

In December 2019, an initial statement emerged from a hospital in Wuhan reporting increased incidences of “viral pneumonia” [1], marking the beginning of a global pandemic and causing the novel disease Covid-19 which is caused by the SARS-CoV-2 virus [2]. After the virus spread rapidly globally and the number of cases increased at an accelerating rate, the situation was declared a pandemic by the WHO on 11 March 2020 [3]. As of 30 May 2021, 192 countries worldwide have been affected by the disease with 169,986,948 confirmed cases and a total of 3,534,435 deaths globally [4]. To limit the spread of the virus and minimize its negative impact, each country developed its strategy to fight the virus through the implementation of measures including cancellation of mass gatherings, closure of public spaces (restaurants, public transport, etc.), ‘stay-at-home’ orders (=‘lockdown’) and protective mask use in public spaces [5]. These measures represented a significant change in everyday life and affected individuals’ usual processes and routines. The ‘Structured Day Hypothesis’ by Brazendale et al. [6] postulated that structured days beneficially regulate obesogenic behaviors. Unstructured days, on the other hand, promote obesogenic behaviors, including lower physical activity (PA), increased sitting time, and other harmful health behaviors. Numerous studies have confirmed this hypothesis during the Covid-19 pandemic, as PA has been repeatedly shown to decrease during the restriction periods [7,8,9,10].

Physical inactivity is associated with many health risks, e.g., type 2 diabetes [11], cardiovascular disease, and certain types of cancer (e.g., lung, prostate, breast, colon, others) [12,13]. It also increases the risk of osteoporosis, fractures in general, and dementia [13] and is associated with a higher incidence of anxiety and depression [14]. This contrasts with the manifold positive aspects of PA: PA reduces the risk of cardiovascular disease and mortality [15] and has a positive long-term effect on weight gain, obesity, coronary heart disease, type 2 diabetes, as well as dementia and Alzheimer’s disease [16]. In addition, PA serves as a protective mechanism against at least 25 chronic diseases [17] and has a favorable/positive impact on emotional and mental health [18,19]. PA has also been shown to reduce inflammatory status, thereby potentially counteracting a SARS-CoV-2 infection [20,21]. Hence, PA should be maintained or even increased, especially against the resistance of social distancing measures during the Covid-19 pandemic.

There is a growing amount of research examining the impact of the Covid-19 pandemic on PA behavior, which is, to the best of our knowledge, currently summarized within three (scoping) reviews. Stockwell et al. [22] reviewed observational cross-sectional, prospective, or retrospective cohort studies with any population, in any setting, published until June 2020, examining changes in PA and sedentary behaviors within the Covid-19 pandemic lockdown. The authors concluded that the majority of studies (64 out of 66) reported decreases in PA and increases in sedentary behaviors during their respective lockdowns across several populations, including children and patients with a variety of medical conditions. López-Valenciano et al. [23] focused on research published until October 2020, without any restriction on study design. The authors concluded a decrease of PA in university students based on 10 studies included in their synthesis, independent of intensity levels. Rossi et al. [24] reviewed evidence of the effects of Covid-19 restrictions on children’s PA and their determinants and concluded that most studies indicated a decrease in PA during the pandemic, based on 84 studies included. The authors also highlighted the importance of age and gender as important determinants.

Today, three reviews exist investigating changes in PA during the pandemic. However, several shortcomings within these reviews should be encountered. First, the existing reviews did not constrain their searches by study design. Most of the included studies used observational and cross-sectional designs. Hence, participants were commonly examined once within the Covid-19 pandemic and were asked about their PA behavior ‘now’ (i.e., at a specific point within the pandemic) as well as retrospectively before the pandemic. This resulted in sometimes long recall periods (i.e., up to over one year if participants were examined in April 2021, for example), raising doubts on the accuracy of these self-reports. In cohort studies, however, results were sometimes obtained from different individuals before and within the pandemic, therefore making causal interpretation impossible. It is recommended to consider only longitudinal designs, investigating the same individuals at different time points, whereas at least one should have been administered before, and one within, the Covid-19 pandemic. Second, the literature suggests that age and gender seem to be important determinants of PA (not only, but also during the Covid-19 pandemic), which has, however, been insufficiently examined until today. Stockwell et al, [22] were the only researchers to differentiate between children, adolescents, and adults. Specific age information is missing in all studies. However, studies have shown that age and gender showed an influence on the expected results: males have been shown to be more active than females throughout their lives [25,26,27], and the amount of PA has been repeatedly shown to decrease over the life span [28,29,30,31]. Hence, a detailed synthesis of different age groups and a distinctive interpretation regarding gender is considered useful. Third, the importance of the objective collection of PA is highlighted several times in the literature [32,33]. However, none of the reviews specifically discussed results of objectively vs. subjectively collected PA data. Advantages of objective methods include the avoidance of bias and a more precise estimate of real PA [34], as subjective measurement methods tend to over- or underestimate PA [35,36]. Hence, the type of measurement should be considered as an additional determinant of interest. Fourth, none of the existing reviews used statistical methods to quantify the Covid-19 induced changes in PA.

In this review, a meta-analytical procedure will be applied to quantitatively estimate the influence of the pandemic’s restrictions, accompanied by a narrative synthesis of results divided by gender and measurement method. Taken together, this systematic review and meta-analysis aimed to examine PA measured before and during the Covid-19 pandemic in longitudinal designs by taking three different determinants (i.e., age, gender, and measurement method) into account.

## 2. Materials and Methods

The present systematic review and meta-analysis is structured according to the updated Preferred Reporting Items for Systematic Reviews and Meta-Analysis (PRISMA) [37]. The full protocol was registered on Prospero (registration number CRD42020226162).

### 2.1. Eligibility Criteria

Primary sources and peer-reviewed articles that addressed PA before and during the Covid-19 pandemic published in English and German were eligible for inclusion in this review and meta-analysis. Studies were selected for eligibility based on the PICOS criteria [38] displayed in Table 1.

### 2.2. Information Sources

Three different electronic databases were used for result retrieval: PubMed, Web of Science, and Scopus. Search terms were adapted to meet specific demands of databases (see Section 2.3). The latest search update was conducted on 6 October 2021.

### 2.3. Search Strategy

A comprehensive search strategy was developed and defined through the discussion of two authors (KW, KK) for all three databases. Using the PICOS search tool [38], search terms consisted of the key search terms ”Covid-19” and “Physical Activity” as well as synonyms and alternative terms thereof, linked by Boolean operators OR and AND:

Covid-19: COVID-19, SARS-CoV-2, COVID 19 pandemic, Corona virus

Physical Activity: Physical Activit*, Physical Exercise*, Home Workout

Database-specific search terms, as well as respective number of hits, can be found in Supplementary Table S1. Identified studies were then transferred to Citavi (Swiss Academic Software GmbH, Wädenswil, Zürich, Switzerland) version (6.5.0.0) for further processing.

### 2.4. Selection Process

First, all duplicates detected by the system were removed. Then, two authors (KW, KK) independently assessed the title and abstract of retrieved studies for eligibility for inclusion. Disagreements were resolved via discussion until consensus was reached among the two screening authors. Afterward, full texts were downloaded into Citavi and checked by the same two authors for their eligibility. If the full text was not available, the relevant authors were contacted by e-mail to gain access to these studies.

### 2.5. Data Collection Process and Data Items

Adapted to the requirements of the current investigation, an Excel spreadsheet was developed. Data extracted from each study included general information (authors, year, country), demographics (sample size, age group), gender), basic information on methods (aim, study design, sampling time points, methods used regarding PA) and results (direct association statistics, central results, and sub findings). For each relationship, relevant effect sizes were retrieved. Data from the eligible studies were extracted by two authors independently (KK, CN) and entered into this spreadsheet. Unclear points were discussed between the two authors. The remaining author (KW) served as a mediator until consensus was reached. After completion, extracted data were double-checked and verified (KW). In case of missing data, the study investigators were contacted for unreported data or additional details. Each study was dummy-coded regarding the examined age-group(s): (1) children and adolescents from 0 to 19 years, (2) adults from 20 to 60 years, and (3) older adults aged over 60 years to account for interindividual, age-related differences in PA levels due to the Covid-19 pandemic.

### 2.6. Risk of Bias and Quality Assessment

To assess the risk of bias across studies, funnel plots were compiled for all age groups using R [39,40]. For the assessment of the risk of bias or methodological quality within individual studies, the Quality Assessment Tool for Before–After (Pre–Post) Studies With No Control Group [41] was used to determine the quality of the studies. The tool rates the risk of bias within a total of 12 questions, including several bias types, with selection bias, reporting bias, and observer bias amongst others. The 12 criteria were assessed and evaluated with yes, no, or other (cannot determine, not applicable, not reported) when the criterion was applicable to the analyzed study or with other when the criterion was not fulfilled, not applicable to the analyzed study, or could not be answered based on the information provided by the review. Risks were rated as good, fair, or poor for each study. Since the National Heart, Lung, and Blood Institute, which provides the tool, does not offer general guidance on how to overall rate a study, the assessment of the individual studies was aligned with the review by Raaijmakers et al. [42]. Each study was considered and evaluated individually. The risk of bias and quality of the study was assessed by two authors (KK, CN), and afterward verified by the remaining author (KW). Any discrepancies were resolved through discussion.

### 2.7. Summary Measures

To perform the meta-analysis, all effect sizes were extracted from the original studies and transformed into correlation coefficients. F-values from ANOVAs, Cohen’s d, or other effect size measures were transformed to correlation coefficients using the online platform psychometrica [43]. If no effect sizes were reported, relevant data were extracted and effect sizes were calculated a posteriori, also using psychometrica. If betas or effect size estimates were reported in the studies and the original correlation coefficients could not be obtained, the betas and effect size estimates were treated as correlation coefficients [32].

### 2.8. Additional Analyses and Synthesis of Results

To gain a basis for meta-analytical interpretation, all effect sizes (correlation coefficients) were Fishers-z-transformed to gain comparable results. First, an ANOVA was calculated to check whether variances are different from zero for age, measurement method, and gender. If differences in variances can be found, age will be added as a moderator. Hence, narrative synthesis, as well as meta-analytical results, are structured according to age groups for better comparison and differentiated summary.

A multilevel approach was chosen to account for multiple effect sizes in single studies. The results were interpreted as follows [36]. Based on empirically derived effect size distribution, correlation coefficient values of 0.12, 0.24, and 0.41 were interpreted as small, medium, and large effects for social psychology studies. The Q-test for heterogeneity [44] is reported to display the amount of heterogeneity. The analysis was carried out using R (version 3.6.1) [39] and the metafor package (version 2.1.0) [45].

### 2.9. Synthesis Method

First, a descriptive (narrative) synthesis of the general characteristics of all included studies is provided. These characteristics include author, year, study country, sample characteristics and participant demographics, PA-related aims, details of type and measurement of PA, sampling time points, PA-related outcomes (e.g., decrease or increase of PA), and their absolute change and facilitate the comparison among studies. Subgroup analysis on age group, gender, and measurement method (self-report, SR, vs. device-based, DB methods), are narratively discussed. Meta-analytical results for gender differences did not reveal a significant overall effect for either gender and are, therefore, not displayed in the current manuscript. Meta-analysis for measurement method was not meaningful due to a low amount of measurement-based effect sizes.

## 3. Results

### 3.1. Study Selection

**Figure 1:**
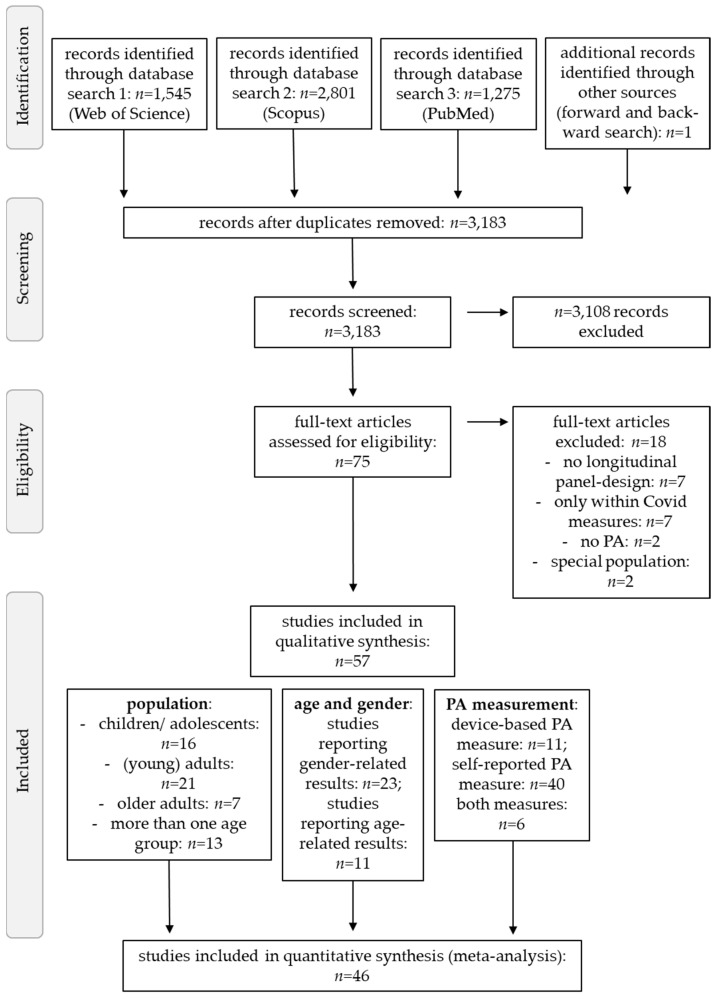
Prisma Flow Chart.

The literature search of the three electronic databases revealed a total of 5621 studies which were downloaded into Citavi. After automatic removal of duplicates, the titles and abstracts of 3182 records remained and were screened for in- and exclusion criteria, resulting in an exclusion of a further 3108 studies. Fifty-seven studies finally met the inclusion criteria and were included in the review. However, 11 of these studies had to be excluded from the meta-analysis due to not reporting (and authors not providing) relevant data for effect size estimation. The main reasons for exclusion were no longitudinal design (e.g., [46,47]), only within Covid-19 measures (e.g., [48]), or no measurement of PA (e.g., [49]). An overview of the entire selection process and the detailed exclusion is presented in Figure 1.

### 3.2. Study Characteristics

Studies were conducted across all continents. Most were from Spain (*n* = 9), followed by the United States of America (*n* = 7), the United Kingdom (*n* = 5), and Japan (*n* = 5). One study collected data in a total of 14 countries [50]. The total sample size is 119,094 (*n*_female_ = 72,680, *n*_male_ = 45,521). Two studies only included female participants [51,52], and two studies did not provide information on gender [53,54]. The number of participants in each study varied from 10 [55] to 60,560 [56] and the age of participants ranged from four [57] to 93 years [58]. Further information on demographic characteristics can be found in Table 2. Due to inclusion criteria, study designs were homogeneous, but studies differed regarding assessment methods for PA. Forty studies solely used self-report (SR) measures, with seven using different versions of the International Physical Activity Questionnaire (IPAQ; [59]), five the Physical Activity Questionnaire for Adolescents (PAQ-A; [60]), three the Global Physical Activity Questionnaire (GPAQ; [61]) and three the MoMo Physical Activity Questionnaire (MoMo-PAQ; [62]). The remaining studies used different questionnaires. Eleven studies solely used device-based (DB) measures. Of these studies, eight used accelerometers, and three used pedometers. Another six studies used both SR and DB measures to assess PA.

PA outcomes were manifold, including total PA in time/week (*n* = 14 studies), time/day (*n* = 7 studies), days/week (*n* = 1 study), and two single questions. Furthermore, step counts were collected in steps/day (*n* = 13 studies), steps/month (*n* = 2 studies) and steps/week (*n* = 1 study). The other PA outcomes most commonly surveyed were moderate to vigorous PA (*n* = 11 studies), moderate PA (*n* = 10 studies), vigorous PA (*n* = 11 studies), and walking (*n* = 9 studies). At this point, it must be mentioned that of the 57 studies, 31 used multiple outcomes.

The majority of studies had two measurement time points (*n* = 43 studies), but there were also studies with more measurement time points (i.e., six studies with three sampling time points, two with four sampling time points). One study used 154 sampling time points. However, device-based measurement methods were used here to measure PA every day [63].

The distance of all before Covid-19 measurement widely varied between studies. Chen et al. [64] performed the earliest before Covid-19 measurement in September 2015, and the latest was carried out by Suzuki et al. [65] on 15 April 2020, who assessed a four-week period before a state of emergency was declared on 16 April 2020. Most before Covid-19 measurements took place in the period between October 2019 and February 2020 (*n* = 32 studies). Regarding the within Covid-19 measurements, the first took place in week 2 of 2020 [66], the latest was carried out in November 2020 [55], while most took place in April 2020 (*n* = 23 studies). In five cases, authors only stated that they had accomplished a before- and within-Covid-19 measurement occasion, but did not reveal whether they had examined their participants within the lockdown or whether the measurements were taken during the pandemic, independent of any restrictions. Thirty-three studies made within Covid-19 measurements during a national lockdown period, six studies had measurements partially during the lockdown, but also collected data beyond this time point, and four studies had measurements only ‘during’ Covid-19 (time with increased restriction, but no lockdown). Another nine studies were conducted in countries where there was no lockdown (Brazil, Japan, South Korea, and Sweden). The date of the lockdowns differed from country to country, so the information applies individually to the respective country and the restrictions in force there [67]. A detailed description of the characteristics of all 57 studies can be found in Table 2.

**Table 2:** Characteristics of included studies.

Author(s) (Year)/Country [Ref]	Sample Characteristics/Population	PA Related Aim	Sample Size, Age (SD)	PA Measurement	Sampling Timepoints	Central/Overall Results	Absolute Change
Aegerter et al. (2021)/Switzerland [68]	Office workers from two Swiss organizations	(...) to quantify the effect of the COVID-19 pandemic on PA levels among Swiss office workers	*n* = 76 (54 female); 42.7 ± 9.2 years	SR: IPAQ-SF	T0: January 2020 T1: April 2020	No sig. change in total PA, walking, MPA, VPA	descriptive study
Al-Musharaf et al. (2021)/Saudi Arabia [51]	Healthy female students or graduates of King Saud University (19–30 years)	(...) to assess lifestyle changes (a.o. PA) from before COVID-19 to during lockdown	*n* = 297 (female); 20.7 ± 1.4 years	SR: GPAQ	T0: February–April 2019 T1: April–May 2020	Total PA: −	Total PA: −126.7 MET-min/week
Alonso-Martinez et al. (2021)/Spain [69]	Preschoolers (4–6 years) from 3 schools in Pamplona	(...) to examine the effects of the COVID-19 lockdown on device-measured PA (...)	*n* = 268 (125 female); 4.28 ± 0.80 years	DB: GENEActiv (accelerometer)	T0: September–December 2019 T1: March–April 2020	Total PA and MVPA: −	Total PA: −43.3 min/day MVPA: −17.0 min/day
Baceviciene and Jankauskiene (2021)/Lithuania [70]	Lithuanian students from a previous, large study	(...) to assess the impact of COVID-19-related lockdown period on PA in university-aged Lithuanian students of both genders (...)	*n* = 230 (182 female); 23.9 ± 5.4 years	SR: LTQE	T0: October 2019 T1: 9 February 2021	Males’ leisure-time PA: −	Males: −20 points Females: −6.01 points
Barone Gibbset al. (2021)/USA [71]	Desk workers, ≥20 h of deskwork and <150 min MVPA per week	(...) to study the longitudinal impact of COVID-19 on lifestyle among desk workers during shelter-at-home restrictions	*n* = 112 (77 female); 45.4 ± 12.3 years	SR: Paffenbarger Physical Activity Questionnaire	T0: 2018–2019 T1: May–June 2020	No sig. change in MPA, VPA, MVPA	MPA: +20 min/week VPA: +/−0 MVPA: +15 min/week
Bartlett et al. (2021)/Australia [72]	Adults (>50 years) who engaged in a public health program targeting dementia risk reduction	(...) to examine longitudinal change on dementia risk factors in a sample of middle-aged and older Tasmanian residents	*n* = 1671 (female 1218); 63.4 ± 7.17 years	SR: min/week for walking, MPA, VPA, TPA	T0: October 2019 T1: April–June 2020	Total PA: +	Total PA: +300.06 min/week
Bronikowska et al. (2021)/Poland [73]	Randomly selected school class from six secondary schools from the urban area of the Wielkopolska region (Greater Poland)	(...) to compare PA levels before and during a pandemic lockdown among adolescent Polish youths (...)	*n* = 127 (66 female); 15.4 ± 0.5 years	SR: Physical Activity Screening Measure	T0: February 2020 T1: June 2020	MVPA WHO rec.: + (*n* = 13), − (*n* = 15) maintained not meeting (*n* = 84) maintained meeting (*n* = 15)	+ MVPA WHO rec.: +2.8 days/week −MVPA WHO rec.: −2.4 days/week maintained not meeting rec.: −0.3 days/week maintained meeting rec.: +0.3 days/week
Buoite Stella et al. (2021)/Italia [74]	Healthy adults (>18 years) in Italy during the COVID-19 lockdown	(...) to investigate changes occurring in daily life and their effects on health during the COVID-19 lockdown (...)	*n* = 400 (277 female); 35 ± 15 years	SR: self-designed online-survey	T0: January 2020 T1: 23–29 March 2020	Step count: −	Ø −4990 steps/day
Chaffee et al. (2021)/USA [75]	Ninth- and tenth-grade students high schools in Northern California	(...) to compare adolescents’ PA behaviors before and after stay-at-home restrictions	*n* = 1006 (623 female); age not reported	SR: Single questionnaire item	T0: March 2019–February 2020 T1: September 2019–September 2020	Total PA: −	descriptive study
Chen et al. (2021)/Sweden [64]	15-year-old adolescents in Sweden	(...) to investigate the impacts of COVID-19 on health behaviors	*n* = 584 (311 female); 15.5 ± 04 years	SR: Web questionnaire	T0: September 2015–June 2019 T1: February 2020–November 2020	PA 60 min/day (days/week): − Weekly duration of LTE: no changes	PA 60 min/day (days/week): −0.2 days/week Weekly duration of LTE: no changes
Cheval et al. (2020)/Switzerland [76]	Participants living in France or Switzerland (76% French)	(...) to assess changes in PA during commuting and leisure during the COVID-19 lockdown (...)	*n* = 110 (76 female); 43 ± 9 years	SR: IPAQ	T1: 30 March 2020 T2: 13 April 2020	PA when commuting, VPA: − Walking, MPA: +	PA when commuting: −16 min/day VPA: −6 min/day walking: +5 min/day MPA: +4 min/day
Curtis et al. (2021)/Australia [77]	Community-based sample of healthy adults from Adelaide, South Australia	(...) to examine changes in recreational PA before and during COVID-19 restrictions in a group of adults in Adelaide, Australia	*n* = 61 (40 female); 41.3 ± 5.8 years	SR: HABITATDB: Fitbit Charge 3	T1: 10–23 February 2020 T2: 14–27 April 2020	LPA, swimming, team sports, boating/sailing: −MVPA: no changecycling: +PA with others in park, running, weights, exercise class, golf, tennis, yoga/pilates/tai chi/qigong, home-based exercise, water activities, PA with others on a beach: no sig. change	LPA: −50 min MVPA: no change; Cycling: +0.35 pt; Swimming: −0.64 pt; Team sports: −0.36 pt; Boating/sailing: −0.13 pt; PA with others in park: −0.32 pt; running: −0.28 pt; weights: −0.31 pt; exercise class: −0.13 pt; golf: −0.03 pt; tennis: +0.04 pt; yoga/pilates/tai chi/qigong: +0.05 pt; home-based exercise: +0.32 pt; water activities: −0.03 pt; PA with others on a beach: −0.11 pt
Di Sebastiano et al. (2021)/Canada [78]	Canadian users (≥18 years) PA tracking app (PAC app)	(...) to investigate changes in the PA of Canadians before and after restrictions in Canada, using data from the ParticipACTION app	*n* = 2338 (2109 female); age range: 18–65 years	DB: PAC app	T1: 10–16 February 2020 T2: 13–19 April 2020	MVPA, LPA, and steps: −	MVPA: −17.5 min/week LPA: −126.4 min/week steps: −5230 steps/week
Ding et al. (2021)/China [79]	Healthy participants (>18 years) from 11 workplaces in Shanghai,	(...) to determine the change in daily steps in response to the lockdown and reopening during the COVID-19 pandemic in China (...)	*n* = 815 (530 female); age range: 20–50+ years	SR: IPAQ-SF DB: WeRun via WeChat (accelerometer)	T0: December 2019–23 January 2020 T1: 24 January–22 March 2020	Step count 1: − (24 January 2020) Step count 2: + (25 January–22 March 2020)	step count 1: −3796 steps/day step count 2: +34 steps/day
Elnaggar et al. (2020)/Saudi Arabia [80]	Healthy adolescents (14–18 years)	(...) to document PA changes in adolescents living in Saudi Arabia	*n* = 63 (29 female); 15.54 ± 1.16 years	SR: PAQ-A	Not reported	PAL: −	PAL: −0.28 PAL
Esain et al. (2021)/Spain [81]	Community-dwelling adults (>65 years) from Getxo (Basque Country)	(...) to analyze the effect of social distancing measures on PA levels in Spanish older adults (...)	*n* = 58 (45 female); 76.24 ± 6 years	SR: MLTPAQ-SF	T0: October 2019 T1: June 2020	Total PA, walking, cleaning: − Exercising or dancing: +	total PA: −2304.74 MET/week walking: −220.00 MET/week cleaning: −210.08 MET/week exercise/dancing: +109.21 MET/week
Folk et al. (2021)/USA [82]	Participants of the EAT 2010–2018 study, who attended middle and high schools in Minnesota in 2009/2010	(...) to understand how PA changed during the time of the COVID-19 pandemic in a diverse sample of emerging adults in the US	*n* = 720 (447 female); 24.7 ± 2 years	SR: Godin-Shepherd Questionnaire	T0: 2018 T1: April–October 2020	Total PA, MVPA, mild PA: −	Total PA: −1.47 h/week MVPA: −0.93 h/week Mild PA: −0.52 h/week
Franco et al. (2021)/Spain [83]	Spanish office employees who participated in the 5th “Healthy Cities” challenge	(...) to analyze how PA among workers has been affected during confinement and whether certain covariates could have influenced the effect of the confinement on the PA among participants	*n* = 297 (148 female); 42.76 ± 7.79 years	SR: IPAQ-SF	T0: October 2019 T1: May 2020	Total PA and MPA: + VPA and walking: no change	Total PA: +463.71 METs MPA: +327.83 METs VPA: +44.32 METs walking: −91.58 METs
Gallego-Gomez et al. (2020)/Spain [84]	Nursing students from the Catholic University of Murcia (Spain)	(...) to identify how PE affected the level of stress of Nursing students before and during the lockdown	*n* = 138 (108 female); 20 years (no SD provided)	SR: Single questionnaire item	T0: 3 February 2020 T1: 24 March 2020 T2: 24 April 2020	PE and median hours of PE: +	Practice of PE: +26 students Median hours of PE/week: +2 h/week
Gilic et al. (2020)/Bosnia and Herzegovina [85]	Adolescents from three counties in B&H attending High school	(...) to evaluate the dynamics of changes in PAL among adolescents from Bosnia and Herzegovina before and during the imposed lockdown	*n* = 688 (322 female); age range: 15–18 years	SR: PAQ-A	T0: 6–12 January 2020 T1: 20–26 April 2020	PAL: −	PAL: −0.67 PAL
Gilic et al. (2021)/Bosnia and Herzegovina [86]	Healthy high school students (<18 years) from 4 counties in B&H	(...) to examine the influence during the COVID-19 pandemic among adolescents from Bosnia and Herzegovina on PALs	*n* = 661 (292 female); age range: 15–18 years	SR: PAQ-A	T0: 6–12 January 2020 T1: 20–26 April 2020	PAL (BL): 48% had sufficient PAL PAL (FU): 24% had sufficient PAL	descriptive study
Giuntella et al. (2021)/USA [87]	Students from the University of Pittsburgh	(...) to examine how PA has evolved during the pandemic compared to pre-pandemic levels and to prior cohorts	*n* = 217 (163 female); 19.22 ± 1.53 years	DB: Fitbit Alta HR	T0: February 2020 T1: April 2020	Step count: − Active hours: −	Step count: −5400 steps/day Active hours: −1.5 h/day
He et al. (2020)/China [88]	Adults from any province of China except Hubei Province (epicenter of the outbreak)	(...) to study the relationships between body weight changes with changes in PA and lifestyle during quarantine	*n* = 339 (181 female); males: 36.4 ± 11.9 years; female: 37.6 ± 12.4 years	DB: Smartphone health software	T0: 23 December 2019–26 January 2020 T1: 27 January–1 March 2020	Step count: − MVPA: −	male steps: −4593 steps male MVPA: −11.8 min/day female steps: −3297 steps female MVPA: −8.6 min/day
Hino et al. (2021)/Japan [66]	Participants (≥18 years) of the YWPP	(...) to analyze the fluctuation of the step counts of citizens in Yokohama city, Japan, in the first half of 2020 compared to the previous year	*n* = 18,817 (9083 female); 53.9 ± 7.7 years	DB: Omron HJ-326F (pedometer)	Week 2–26 in 2019 and 2020 T0: Week 15–21, 2019 T1: Week 15–21, 2020	Step count year-on-year ratio: −	descriptive study
Koohsari et al. (2021a)/Japan [89]	Company workers (20–59 years)	(...) to examine the changes in PA of company workers during the COVID-19 outbreak in Japan (...)	*n* = 2466 (1212 female); 39.6 ± 10.7 years	SR: GPAQ	T0: February 2019 T1: July 2020	Total PA, VLPA: − VWPA, MWPA, TPA, MLPA: no sig. change	VWPA: −0.02 h/day MWPA: −0.05 h/day TPA: −0.04 h/day VLPA: −0.05 h/day MLPA: −0.04 h/day Total PA: −0.20 h/day
Martinez-de-Quel et al. (2020)/Spain [90]	Students (>18 years); at University Madrid, Léon, Vigo, or University Isabel I or others	(...) to show the impact that the lockdown period had on the PA levels to a sample of Spanish individuals due to COVID-19	*n* = 161 (60 female); 35 ± 11.2 years	SR: MLTPAQ	T0: 16–31 March 2020 T1: 30 April and 11 May 2020	Total PA: −	Total PA: - 3462.2 MET min/Week
McCarthy et al. (2021)/United Kingdom [58]	Individuals (≥14 years) in the UK registered with BetterPoints (free, publicly available, smartphone-based program)	(...) to explore patterns of tracked activity in the UK before, during, and after the COVID-19 restrictions and to explore variations by demographic characteristics	*n* = 5395 (3274 female); 41.02 ± 12.2 years	DB: BetterPoints smartphone app	T0: 22 January 2020 T1: 11 March 2020 T1.1: 18 March 2020 T1.2: 25 March 2020 T1.3: 13 May 2020 T2: 17 June 2020	Total PA: −	Total PA (BL to T1): −30 min/week Total PA (BL to T2): −67 min/week Total PA (BL to T3): −95 min/week Total PA (BL to T4): −69 min/week Total PA (BL to T5): −71 min/week
Medrano et al. (2020)/Spain [91]	Cohort of children of the MUGI project in Navarra (8–16 years)	(...) to examine the effects of the COVID-19 confinement on lifestyle behaviors in a cohort of Spanish children (...)	*n* = 113 (55 female); 12.0 ± 2.6 years	SR: YAP	T0: September–December 2019 T1: March–April 2020	Total PA: −	Total PA: −91 min/day
Mishra et al. (2021)/USA [55]	Community-dwelling older adults (≥75 years) or aged 65 years older with a high risk of falling	(...) to examine changes from pre- to post-pandemic in mobility performance, including walking characteristics (...)	*n* = 10 (4 female); 77.3 ± 1.9 years	DB: PAMSys (pendant sensor)	Not reported	daily walking duration and step count: − LPA and MVPA: no change	walking duration: −52.2% Step count: −3256 steps/day LPA: −0.3 min/day MVPA: −3.7 min/day
Miyahara et al. (2021)/Japan [92]	Elderly people residing in Asakita Ward, Hiroshima City	(...) to elucidate how much self-restraint from activity by the elderly with diseases reduces PA	*n* = 13 (11 female); 77.5 ± 3.5 years	DB: HJA-750C OMRON (accelerometer)	T0: October 2019 T1: April 2020	Steps, AT, MPA, MLAPA, LPA, LWAPA, LLAPA, total PA: − Walking, LA, MWAPA: no change	Steps: −2236, 1 steps/d; AT: −98.4 min/d; MPA: −1.8 METs h/d; MLAPA: −1.3 METs h/d; LPA: −2.4 METs h/d; LWAPA: −0.3 METs h/d; LLAPA: −2.1 METs h/d; Total PA: −4.2 METs h/d; Walking: −0.1 METs; LA: no change; MWAPA: −0.5 METs h/d
Munasinghe et al. (2020)/Australia [63]	Young people from the general population (13–19 years) of Western Sydney	(...) to investigate whether the physical distancing policies were associated with changes in PA in the state of New South Wales (Australia)	*n* = 582 (465 female); median age: 17 years	SR: PACE + Adolescent PA Measures DB: Smartphone sensors	T0: 8 November 2019–23 March 2020 T1: 24 March–19 April 2020	Total PA: − Step count: − MBAR: −	descriptive study
Nigg et al. (2021)/Germany [93]	Children and adolescents (4–17 years) living in Germany	(...) to investigate whether participants living in areas with higher population density demonstrate less positive PA changes	*n* = 1711 (852 female); 11.34 ± 4.06 years	SR: MoMo-PAQ	T0: August 2018–March 2020 T1: 20 April–1 May 2020	Active days/week, daily life PA: + sports-related PA: −	Active days: +0.47 days/week Sport-related PA: −68.33 min/week Daily life PA: +37.74 min/day
Nyström et al. (2020)/Sweden [57]	Preschoolers (3–5 years) from Stockholm County and County of Östergötland	(...) to assess how movement behaviors have been affected in Swedish preschool children during the COVID-19 pandemic	*n* = 100 (42 female); 4.0 ± 0.5 years	SR: Self-developed questionnaire DB: ActiGraph	T0: March–May 2019 T1: May–June 2020	Total PA, time spent outside weekdays and weekends: +	Total PA: +53 min/day Time spent outside (weekdays): +124 min/day Time spent outside (weekend): +68 min/day
Obuchi et al. (2021)/Japan [94]	Subscribers to a life insurance plan from a private insurance service in Japan	(...) to determine the effects of self-restraints on daily walking parameters	*n* = 3901 (2969 female); 60.3 ± 28.9 years	DB: Smartphone application	T0: 2 March–15 June 2019 T1: 2 March–15 June 2020	Step count: −	steps: −1000 steps/week
Okely et al. (2021)/14 countries [50]	Children (3–5 years) of the SUNRISE study	(...) to examine how the COVID-19 pandemic influenced PA among preschoolers (...)	*n* = 948 (466 female); 5.2 ± 0.6 years	SR: Parent/Caregiver survey	T0: April 2019–March 2020 T1: May–June 2020,	No significant changes	Total PA: +17 min/day MVPA: −5 min/day
Okely et al. (2020)/United Kingdom [95]	Participants rom the Lothian Birth Cohort 1936 (LBC1936) study, all born in 1936	(...) to examine changes in PA among older people during COVID-19 lockdown, and if participant characteristics were related to more positive or negative changes during the lockdown	*n* = 137 (66 female); 84 years	SR: Single questionnaire item	T0: 2017–2019 T1: 27 May 2020	Total PA: − Minimal PA: +	descriptive study
Ong et al. (2020)/Singapore [96]	Young adults (21–40 years) working in the Central Business District in Singapore	(...) to characterize how COVID-19-associated mobility restrictions shifted PA patterns from previously established baselines	*n* = 1824 (941 female); 30.94 ± 4.62 years	DB: Fitbit API	T0: 2–22 January 2020 T1: 17 March–6 April 2020 T2: 7–27 April 2020	Step count and MVPA: −	Steps WD (T0–T1): −1548 steps Steps WE (T0–T1): −1569 steps MVPA WD (T0–T1): −4.1 min MVPA WE (T0–T1): −4.9 min Steps WD (T0–T2): −4060 steps Steps WE (T0–T2): −3560 steps MVPA WD (T0–T2): −13.2 min MVPA WE (T0–T2): −13.7 min
Park et al. (2021)/South Korea [97]	Adults (>18 years) in South Korea	(...) to investigate the changes in health-related behaviors and outcomes pre-COVID-19 and during COVID-19 (...)	*n* = 834 (380 female); 23.7 ± 6.0 years	DB: Data from smartphone health app	T0: January 2019–February 2020 and May 2020 T1: June, July, & October 2020 T2: March, April, & August 2020 T3: September 2020	Step count: −	Step count: −935 steps (mean decrease) T0–T1: −539 steps T0–T2: −1131 steps T0–T3: −1136 steps
Perez et al. (2021)/Spain [98]	Nondisabled frail older adults from the +ÀGIL Barcelona project	(...) to describe PA changes due to mobility restrictions in community-dwelling, frail older persons from Barcelona, who had not been diagnosed with COVID-19	*n* = 98 (65 female); 82.4 ± 6.1 years	SR: BPAAT	T0: May 2019 T1: May 2020	Total PA: − sufficient PA: −	Total PA: −1.1/8 points sufficient PA: −32.2%
Riberiro de Lima et al. (2021)/Brazil [52]	Physically inactive females (50–70 years)	(...) to analyze the effects of this pandemic period on PA in women aged 50 to 70 years	*n* = 34 (female); 58.5 ± 6.0 years	SR: MBQO	T0: January–February 2020 T1: June–July 2020	Domestic PA, free time PA: − Sports PA, total PA (MBQO score): no sig. changes	Domestic PA: −5.8% Free time PA: −83.2% Sports PA: −7.1% Total MBQO score: −19.9%
Richardson et al. (2020)/United Kingdom [99]	Older adults (≥70 years) recruited throughout the UK by self-selection, through online advertisements	(...) to examine the impact that COVID-19 measures in the UK, had on individuals aged 70 and over in terms of their PA levels	*n* = 117 (65 female); 75 ± 4 years	SR: IPAQ-E	T0: 11 March–28 March T1: 4 April T2: 18 April T3: 2 May	Total PA: no sig. change	T0–T1: +87 MET-minutes T0–T2: +185 MET-minutes T0–T3: +109 MET-minutes
Romero-Blanco et al. (2020)/Spain [100]	First- to fourth-year health sciences students	(...) to analyze the PA university students did before and during the lockdown and to look at changes resulting from sociodemographic characteristics	*n* = 213 (172 female); 20.5 ± 4.56 years	SR: IPAQ-SF	T0: 15–30 January 2020 T1: 1–15 April 2020	Days of VPA and MPA, total minutes of PA: +	Days of VPA: +1.21 days Days of MPA: +1.41 days Total minutes of PA: +159.87 min/week
Sanudo et al. (2020)/Spain [101]	College students from different schools in Seville	(...) to determine to what extent PA changed during the COVID-19 lockdown	*n* = 20 (9 female); 22.6 ± 3.4 years	SR: IPAQ DB: Xiaomi Mi Band 2 (accelerometer)	T0: February 2020 T1: 24 March–3 April 2020	walking time, MPA, VPA, MVPA, step count: −	walking time: −335 min/week MPA: −263 min/week VPA: −188 min/week MVPA: −451 min/week Step count: −5771 steps/day
Savage et al. (2021)/United Kingdom [102]	University students in the UK	(...) to investigate the changes in PA in university students from before to after the COVID-19 pandemic	*n* = 255 (193 female); 18.97 years (no SD provided)	SR: EVS	T0: 14 October–4 November 2019 T1: 19 October–1 November 2020	MVPA: −	MVPA: −50 min/week
Savage et al. (2020)/United Kingdom [103]	Students of a UK University who were part of the Student Health Study	(...) to investigate changes in PA in UK university students before, in week one, and five weeks into the lockdown (...)	*n* = 214 (154 female); 20.0 years (no SD provided)	SR: EVS	T0: October 2019 T1: January 2020 T2: March 2020 T3: April 2020	MVPA: −	MVPA: −30 min/week
Schmidt et al. (2020)/Germany [104]	Children and adolescents (4–17 years) living in Germany	(...) to investigate how PA in children and adolescents in Germany changed from before to during the COVID-19 lockdown	*n* = 1711 (852 female); 11.34 ± 4.06 years	SR: MoMo-PAQ	T0: August 2018–March 2020 T1: 20 April–1 May 2020	Days active, adherence to the WHO PA guidelines, nonorganized sports, playing outside, gardening, housework, total HA: + Organized sports, total amount of sports: − walking and cycling: no sig. change	days active: +0.44 days/week adherence to PA guidelines: descriptive organized sports: −28.5 min/day nonorganized sports: +17.7 min/day total amount of sports: −10.8 min/day playing outside: +21.4 min/day walking and cycling: +1.8 min/day gardening: +6.7 min/day housework: +4.0 min/day total amount of HA: +36.2 min/day
Sekulic et al. (2020)/Croatia [105]	Adolescents attending high school from Split, Dalmatia County	(...) to evaluate the level of changes in PALs among adolescents from southern Croatia (...)	*n* = 388 (126 female); 16.4 ± 1.9 years	SR: PAQ-A	T0: February 2020 T1: 5–10 April 2020	PAL: −	PAL: −0.32
Suzuki et al. (2020)/Japan [65]	Randomly selected patients (>65 years) from the patient database of a rehabilitation hospital in Kure city,	(...) to understand the impact of public health restrictions on community-dwelling older adults concerning the changes in PA (...)	*n* = 165 (115 female) 78.6 ± 8.0 years	SR: PAQ-EJ	T0: 20 March–15 April 2020 T1: 16 April–13 May 2020	less active group: transportation, light exercise/sports activity, moderate/strenuous exercise/sports, light housework, moderate/heavy housework, total PA: − resistance exercise/sports, labor: no change more active group: light exercise/sports activity, light housework, moderate/heavy housework, total PA: + transportation, moderate/ strenuous exercise/ sports, resistance exercise/sports, labor: no change	less active group: transportation: −3.0 MET h/week light exercise/sports: −6.0 MET h/week moderate/strenuous exercise/sports: −4.1 MET h/week light housework: −4.7 MET h/week moderate/heavy housework: −2.4 MET h/week total PA: −23 MET h/week more active group: light exercise/sports activity: +2.9 MET h/week light housework: +3.7 MET h/week moderate/heavy housework: + 6.9 MET h/week total PA: +24.7 MET h/week
To et al. (2021)/Australia [56]	Registered members of the 10,000 Steps program	(...) to investigate changes in PA reported through the 10,000 Steps program during the COVID-19 pandemic	*n* = 60,560 (40,583 female); age range: 18–45 years	SR: manually registered steps DB: steps automatically synced from activity trackers	Ongoing between 1 January 2018, and 30 June 2020 T1: 1 December 2019 T2: 25 January 2020 T3: 5 February 2020 T4: 2 March 2020 T5: 8 May 2020	Step count (T1, Ø of 30 d; T2, Ø of 7 d; T3, Ø of 7 d; T4, Ø of 7 d, Ø of 30 d): − Step count (T5, Ø of 7 d; T5, Ø of 30 d): + No change: T1, Ø of 7 d; T2, Ø of 30 d; T3, Ø of 30 d	T1: steps: −99 steps (Ø of 7 d); −174 steps (Ø of 30 d) T2: steps: −144 steps (Ø of 7 d); −39 steps (Ø of 30 d) T3: steps: −69 steps (Ø of 7 d); +13 steps (Ø of 30 d) T4: steps: −325 steps (Ø of 7 d); −485 steps (Ø of 30 d) T5: steps: +130 steps (Ø of 7 d); +356 steps (Ø of 30 d)
Wang et al. (2020)/China [106]	Participants (≥40 years) from Step Study 2018 in Changsha, China	(...) to determine if there was any change in daily steps and examine risk factors for frequent low daily steps during the COVID-19 epidemic	*n* = 3544 (1226 female); 51.6 ± 8.9 years	DB: Accelerometer sensor in the smartphone via WeChat	T0: 22 December 2019–20 January 2020 T1: 22 January–20 February 2020	Step count: −	Step count: −2657 steps/d
Wilson et al. (2021)/USA [107]	Undergraduates enrolled in general health and wellness classes	(...) to examine the impact that COVID-19 had on PA among college students by comparing temporal changes in PA over the course of the US spring academic semester	*n* = 187 (128 female); 20.9 ± 1.5 years	SR: GPAQ	T0: January 2020 T1: April 2020	MPA, VPA, MET, active travel, strength training: −	male: MPA: −60 min/week VPA: −75.7 min/week MET: 845.7 min/week AT: −174 min/week ST: −0.6 days/week female:MPA: −52.7 min/week VPA: −18.8 min/weekv MET: 361.1 min/week AT: −266.7 min/week ST: −0.1 days/week
Woodruff et al. (2021)/Canada [108]	Participants (≥18 years) who regularly wear activity trackers	(...) to investigate how PA changed within the first month of the COVID-19 pandemic	*n* = 121 (96 female); 36.2 ± 13.12 years	SR: Data from wearable activity tracker filled into a calendar	T0 and T1 were determined for each participant individually (M T1 = 16 March 2020, SD = 4.7 days, range = 13–31 days).	Step count: −	steps: −1012 steps/day
Wunsch et al. (2021)/Germany [109]	Children and adolescents (4–17 years) living in Germany	(...) to examine the direct influence of the COVID-19 lockdown on PA in a nationwide child and adolescent sample in Germany	*n* = 1711 (961 female); 10.36 ± 4.04 years	SR: MoMo-PAQ	T0: August 2018–March 2020 T1: April 2020	days/week with at least 60 min of PA (4–10-year-olds, 11–17-year-old girls): + 11–17-year-old boys: no change	days/week with at least 60 min of PA: 4–10-year-old boys: +0.65 days/week 11–17-year-old boys: +0.18 days/week 4–10-year-old girls: +0.65 days/week 11–17-year-old girls: +0.41 days/week
Yang and Koenigstorfer (2020)/USA [110]	Healthy U.S. residents (18–65 years)	(...) to investigate the change in PA during the Covid-19-caused lockdown with a focus on PA app use and the features of these apps	*n* = 431 (211 female); 39.1 ± 10.6 years	SR: IPAQ-SF	T0: 12 March–17 March 2020 T1: 4 weeks after restrictions implemented, different in states; Ø: 43.7 (±4.7) days	MPA, VPA, active PA, PA MET: − walking: no sig. change	MPA: −10.4 min/day VPA: −8.5 min/day active PA: −23.4 min/day PA MET: −605.1 MET min/week walking: −4.5 min/day
Zenic et al. (2020)/Croatia [53]	Adolescents attending high school from Split-Dalmatia County	(...) to explore the changes in PALs that occurred because of COVID-19 and social distancing measures in adolescents from Croatia (...)	*n* = 823 (no gender provided); 16.5 ± 2.1 years	SR: PAQ-A	T0: 1–10 March 2020 T1: 5–10 April 2020	PAL: −	PAL: −0.34
Zheng et al. (2020)/China [54]	No information provided	(...) to investigate PA levels in Hong Kong young adults during the COVID-19 pandemic and the changes after the COVID-19 outbreak	*n* = 70 young adults; age not reported	SR: IPAQ	T0: 2019 T1: not provided	MPA, VPA, walking: −	MPA: −5.7 min/day VPA: −3.5 min/day walking: −19.9 min/day
Znazen et al. (2021)/Saudi Arabia [111]	Saudi Arabian university students (18–22 years)	(...) to determine the impact of COVID-19-induced home confinement on lifestyle behaviors	*n* = 144 (90 female); 19.3 ± 1.8 years	SR: SLIQ	T0 & T1: Before and during 75 days of confinement (not specified when)	Total PA: −	Total PA: −3.08 points (activity raw score)

Note: Instruments: BPAAT: Brief Physical Activity Assessment Tool; EVS: Exercise Vital Sign Questionnaire; GPAQ: Global Physical Activity Questionnaire; IPAQ: International Physical Activity Questionnaire; IPAQ-E: International Physical Activity Questionnaire for the Elderly; IPAQ-SF: International Physical Activity Questionnaire Short Form; LTQE: Leisure Time Exercise Questionnaire; MBQO: Modified Baecke Questionnaire for Older Adults; MLTPAQ: Minnesota Leisure Time PA Questionnaire; MLTPAQ-SF: Minnesota Leisure Time PA Questionnaire Short Form; MoMo-PAQ: MoMo Physical Activity Questionnaire; PAC app: ParticipACTION app; PAQ-A: Physical Activity Questionnaire for Adolescents; PAQ-EJ: Physical Activity Questionnaire for Elderly Japanese; SLIQ: Simple Lifestyle Indicator Questionnaire; YAP: Youth Activity Profile Questionnaire; YWPP: Yokohama Walking Point Program.; Results: AT: activity time; HA: forms of habitual PA besides sports; LA: living activity; LLAPA: light intensity living activity physical activity; LPA: light physical activity; LTE: Leisure-Time Exercise; LWAPA: light intensity walking activity physical activity; MBAR: motion-based activity recognition; MET: metabolic equivalent; MLAPA: moderate intensity living activity physical activity; MLPA: moderate leisure-related physical activity; MPA: moderate physical activity; MVPA: moderate to vigorous physical activity; MWPA: moderate work-related physical activity; MWAPA: moderate intensity walking activity physical activity PA: physical activity; PAL(s): physical activity level(s); PE: Physical Exercise; TPA: transport-related physical activity; VLPA: vigorous leisure-related physical activity; VPA: vigorous physical activity; VWPA: vigorous work-related physical activity; pt = points; time points: T0: baseline measurement; Tn: follow up measurement(s).

### 3.3. Risk of Bias within Studies

By quantifying the risk of bias within studies by the Quality Assessment Tool for Before–After (Pre–Post) Studies With No Control Group [41], 26 studies were rated “good”, 14 studies were rated “fair”, and 17 studies was rated as “poor”. The main weaknesses were that the loss to follow-up after baseline was more than 20% and that the outcome measures of interest were not taken multiple times before the measurement. In most studies, no information was provided on “Were the people assessing the outcomes blinded to the participant?” (Question 8, whether the intervention was conducted at a group level (e.g., a whole hospital, a community, etc.), and if the statistical analysis considered the use of individual-level data to determine effects at the group level (Question 12). Therefore, these were always rated as not applicable (NA). Another reason for the “poor” rating included invalid measurement methods because data was self-reported. The complete risk of bias assessment can be found in Table 3.

### 3.4. Risk of Bias across Studies

**Figure 2:**
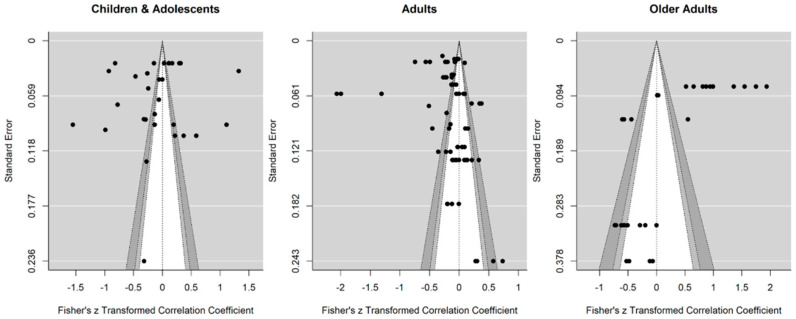
Funnel plots for publication bias between studies.

Publication bias across studies was assessed using funnel plots for all age groups. For better comparison, the reference line was centered to zero. Visual inspection of funnel plots (Figure 2) indicated high publication bias, as small studies with lower power are lacking, especially in children and adolescents and adults. This may result from the fact that most researchers used nationwide datasets to add Covid-19 related data with high relevance for policy makers. Hence, data needs to be interpreted with caution.

### 3.5. Results of Individual Studies

**Figure 3:**
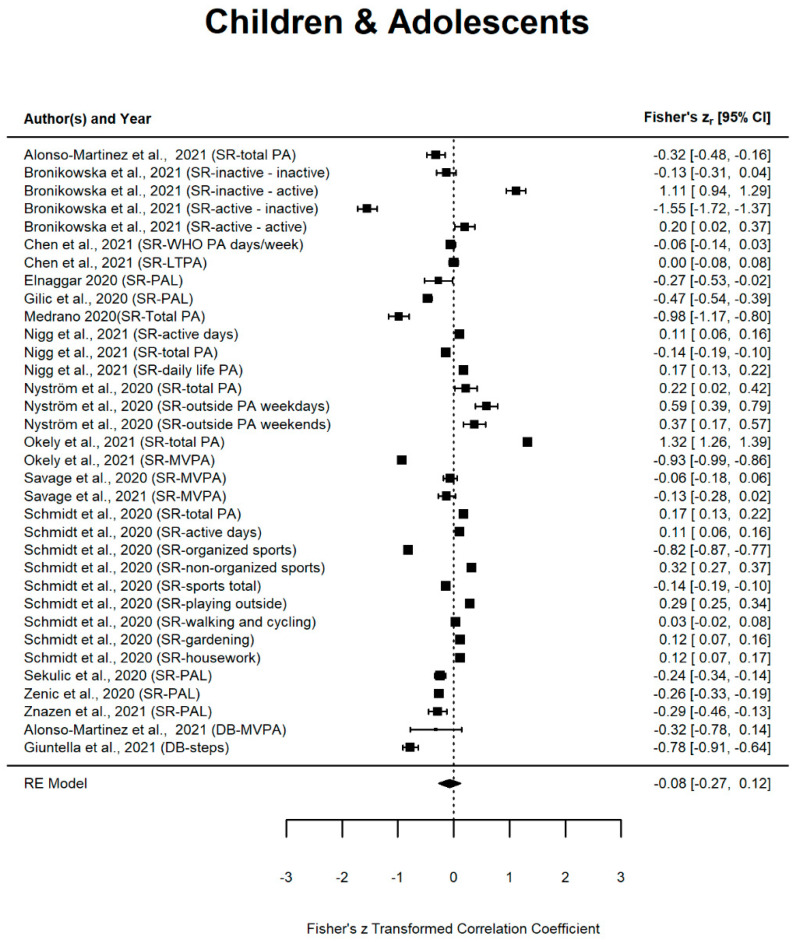

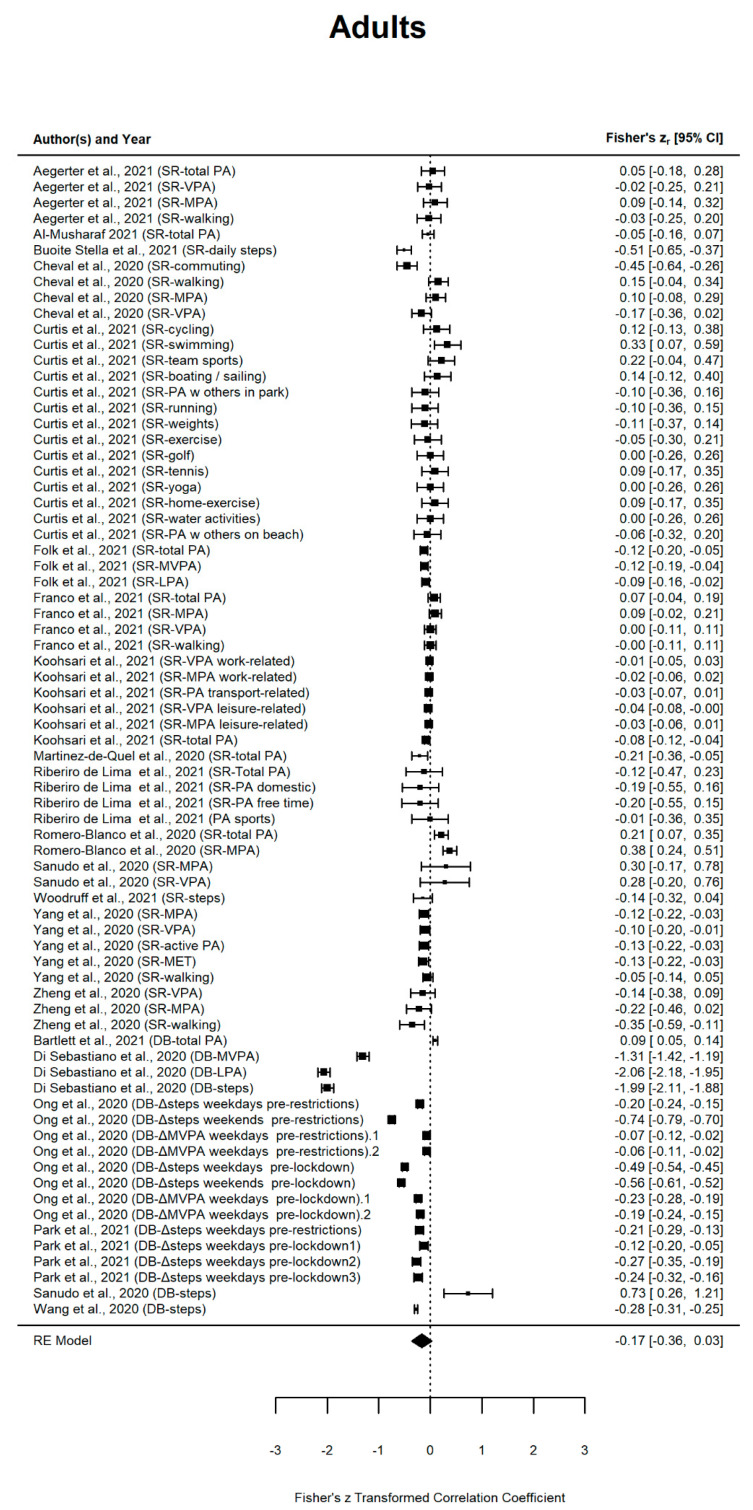

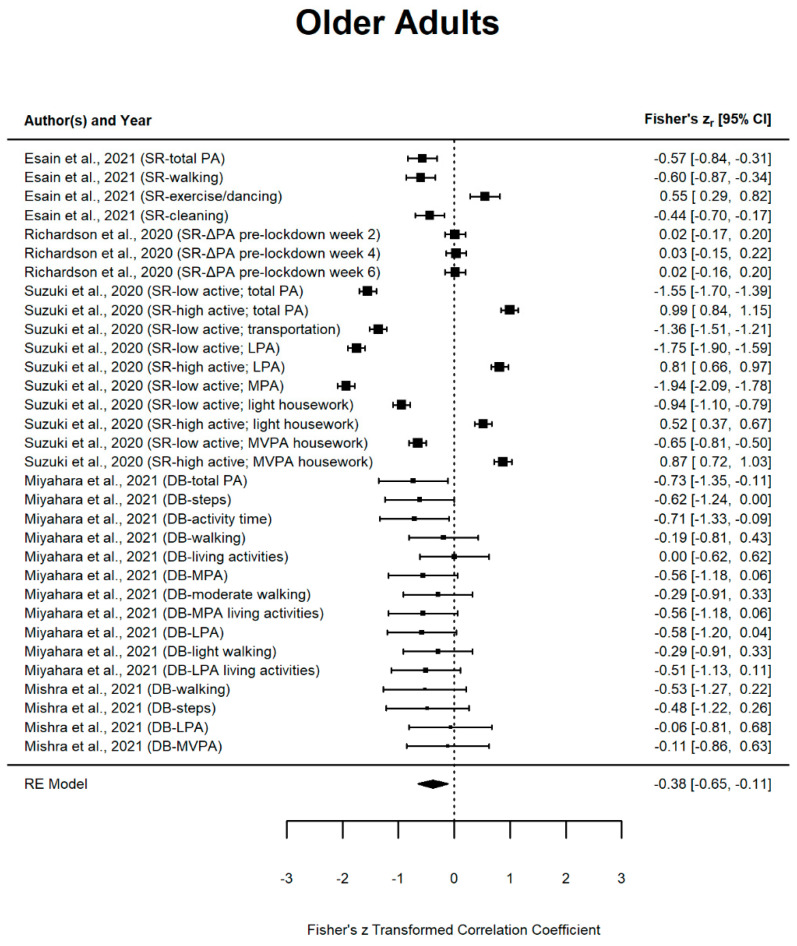
Forest plots of PA changes during Covid-19 for Children and Adolescents, Adults, and Older Adults. Studies are presented in alphabetical order, with those representing more than one age group being presented at the lower part of each forest plot, followed by studies using device-based measurements. Abbreviations: SR: self-reported, DB: device-based; PA: physical activity, LTPA: leisure-time physical activity, WHO: World Health Organization, LPA: low physical activity, MPA: moderate physical activity, VPA: vigorous physical activity, MVPA: moderate-to-vigorous physical activity, PAL: physical activity levels. *Note***:** The 46 studies included in the quantitative meta-analysis with the respective reference numbers can be found in Table 2.

The majority of studies noted a significant reduction in PA during the Covid-19 lockdown (*n* = 32). In contrast, only few studies found an increase in PA (*n* = 5) [57,72,84,100,109] while six studies failed to detect any changes in PA [50,51,68,71,86,99]. Fourteen other studies showed both, a decrease and an increase in PA, depending on the PA characteristics under study. More detailed information on the results of individual studies can be found in Table 2 and Figure 3.

### 3.6. Results of Synthesis

First, results of all studies (N = 57) are presented narratively and are discussed regarding measurement- and gender-related differences, divided by age groups. All results are presented by age group. For this purpose, three groups were classified in advance: children and adolescents (0 to 19 years), adults (20 to 65 years), and older adults (over 65 years).

#### 3.6.1. Children and Adolescents

Overall results. Overall, 25 studies examined PA in children and/or adolescents. 16 of them investigated solely children and/or adolescents and will be summarized below, while nine also included other age groups in their research. Four studies [87,102,103,111], contained participants between 0 and 65 years (age group 1 and 2) and five studies [56,58,78,94,108] included all age groups (age group 1 to 3). Results of studies including different age groups are summarized below. Therefore, the synthesis of the studies that examined solely children and adolescents is presented first, followed by the studies [111] that included several age groups.

The 16 studies examined a total of 11,484 (5587 females) participants (one did not provide information on gender [53]) with an age range from four to 18 years [85,86]. Alonso-Martinez et al. [69] were the only researchers who solely used device-based measurements, and Munasinghe et al. [63] and Nyström et al. [57] used both, SR and DB measurement methods. The remaining studies used SR measurement methods only.

Half of the studies (8/16) found a reduction in PA parameters. Four of these noted this reduction in PA levels [53,80,85,105]. Another four studies concluded that the total PA of the participants studied significantly decreased during the Covid-19 pandemic [63,69,75,91]. In addition, moderate-to-vigorous PA (MVPA) [69] and the number of steps significantly decreased [63]. The results of Chen et al. [64] also showed a significant reduction solely in MVPA but did not reveal any changes in leisure-time exercise.

The results of three studies showed both, an increase and a decrease in PA during the Covid-19 pandemic. Schmidt et al. [104] and Nigg et al. [93] (2021) recognized a reduction in sports-related PA on one hand and a significant increase in active days and habitual PA (playing outside, walking, and cycling, gardening, housework) on the other hand. Bronikowska et al. [73] revealed that more than half of their sample had reduced their PA, a little more than a third had increased their PA, and about one-tenth had kept it alike. In contrast, Nyström et al. [57] revealed an increase in PA and in time spent outside in children. The study by Wunsch et al. (2021) [109] also showed an increase in PA among children and especially among female adolescents. One study reported results only descriptively [86] and another did not detect significant changes in PA [50].

Results based on measurement methods (DB vs SR). Investigating the results of children and adolescents regarding the measurement methodology, no clear statement can be made. The only study using a DB measurement method showed a reduction in PA [69]. Two studies used both, SR and DB methods and revealed a reduction in PA on the one hand [63] and an increase on the other [57]. The remaining studies all used SR methods and came to mixed conclusions.

Results based on gender. Eight studies investigated the difference between gender. Three of them [91,93,104] did not reveal any differences in PA by gender and one study [86] did only report descriptive results. The remaining studies drew a heterogeneous conclusion. Chen et al. [64] revealed a significant reduction in observed PA only for females while, in contrast, Elnaggar et al. [80] and Sekulic et al. [105] found this significant reduction only for males. The latter authors additionally detected a higher PA level at both measurement time points in boys. Gilic et al. [85] found a significant reduction in PA in both boys and girls, and also noted that boys had significantly higher PA levels than girls.

#### 3.6.2. Adults

Overall Results. A total of 34 studies examined changes in PA among 20- to 65-year-old participants. In 21 studies, the sample consisted solely of individuals in this age group, and 13 additional studies also included individuals from other age groups. The synthesis of the research with age groups 1 and 2 (*n* = 4) as well as all three age groups (*n* = 5) has already been stated and analyzed above. In addition, four studies included participants from age groups 2 and 3 (between the ages of 20 and over 65). These results will be presented below. Overall, 13 studies had “good”, six had "fair", and another six had “poor” methodological quality or risk of bias within studies.

Studies investigating adults between 20 and 65 years included a total of 9801 (5530 female) participants (one study did not provide information about gender distribution [54]) with a mean age ranging from 20 [84] to 45.4 [71].

Most studies showed a significant decrease in PA (*n* = 10 studies). This was most frequently observed in steps [74,88,96,97,101], followed by moderate PA (MPA) [54,82,101,107], vigorous PA (VPA) [54,89,101,107] and total PA [82,89,90,107]. Three studies found a significant decline in MVPA [82,88,96]. Additionally, Sanudo et al. [101] and Zheng et al. [54] revealed that walking behavior was decreased, as well as active travel and strength training. Martinez-de-Quel et al. [90] stated that significantly more participants were physically inactive during the Covid-19 pandemic compared to before the pandemic.

Yang and Koenigstorfer [110] revealed a significant decrease in MPA and VPA but did not detect any significant change in walking. Furthermore, Baceviciene et al. [70] reported a significant decline in leisure-time PA for males. Contrary to these findings, two studies revealed that PA increased during the Covid-19 pandemic. On the one hand, there was a significant increase in the practice of physical exercise and the median hours of physical exercise per week [84]. On the other hand, the number of days on which participants engaged in MPA and VPA as well as total PA was found to have increased significantly [100]. Likewise, according to Franco et al. [83], both total PA and MPA increased significantly within the pandemic; however, this research group did not find significant differences for VPA and walking. The results of Cheval et al. [76], Curtis et al. [77], and Ding et al. [79] showed both a reduction and an increase in the measured levels of PA. The latter results represent a special case, as the lockdown initially led to a significant reduction in steps; however, this was followed by a significant increasing trend. The results of the first showed a significant decrease in PA when commuting, but also a significant increase in time spent walking and MPA during leisure time. Curtis et al. [77] concluded that LPA and recreational PA such as swimming, team sports, and sailing decreased significantly. These authors, however, detected a significant increase in cycling, while MVPA and the other recreational activities showed no significance in any direction. Aegerter et al. [68], Al-Musharaf et al. [51], and Barone Gibbs et al. [71] did not detect significant changes in PA, or obtained negligible evidence respectively.

Results based on measurement methods (DB vs SR). Three studies used solely DB methods to examine PA [88,96,97], and three others used DB and SR methods concomitantly [77,79,101]. The remaining used SR measurement methods. Regarding the PA measurement methods, it is noticeable that the PA patterns collected with DB methods (almost) all showed a significant decline. Only for MVPA [77] was no change detected. On the other hand, measurements with SR methods revealed very heterogeneous results regarding an increase in PA, decrease in PA, and no change in PA.

Results based on gender. Six investigations reported results regarding differences between gender. Of these, one found a significant reduction of PA in men only [70], one in women only [100], and one detected marginal differences, in that women had a higher increase in MPA and walking than men [76]. The remaining three studies did not detect any differences or found that the pandemic had the same effect for both genders [83,97,107].

#### 3.6.3. Older Adults

Of the 57 included studies, 16 investigated PA before and during the Covid-19 pandemic in participants aged 65 and older. Seven of these studies dealt exclusively with people over 65, while a further nine also included other age groups (see Section 3.6.4). Together, they investigated 598 (371 female) older adults with a mean age between 76.24 [81] and 84 years [95]. Overall, two studies received a good QA rating, three a fair rating, and another two a poor rating.

Three studies concluded that PA decreased significantly during the Covid-19 pandemic [92,95,98]. They examined total PA, MPA, moderate-intensity living activity PA, LPA, moderate-intensity walking activity and living activity PA, daily activity time, and the number of steps per day [92]. In addition, a higher number of participants reported minimal PA [95], and a decline in reporting sufficient PA [98] was noted. Mishra et al. [55] also found a significant decline in walking duration and daily step count, but they could not detect a significant change in LPA and MVPA. Esain et al. [81] and Suzuki et al. [65] found evidence for both decreasing, and increasing PA during the Covid-19 pandemic. The former found a significant decline in total PA, walking, and cleaning, and a large increase in exercising or dancing. The second stated that nearly half the sample decreased their PA while a little more than 20% increased their PA, whereas the remaining participants maintained their PA levels. The largest reduction in PA took place in light exercise or sports and in moderate or strenuous exercise or sports. In contrast, the largest increase in PA was also found in light exercise or sports activity and in moderate or heavy housework.

Results based on measurement methods (DB vs SR). Mishra et al. [55] and Miyahara et al. [92] used DB methods and either revealed a reduction of PA or failed to detect any significant changes; all other studies used SR methods. In contrast, these studies that used SR measurement methods showed a heterogeneous picture with both decreases and increases of PA during the pandemic. For example, Richardson et al. [99] found negligible evidence that SR PA levels had increased.

Results based on gender. None of the studies reported outcomes related to age or gender. Richardson et al. [99] merely stated that the results applied to both genders.

#### 3.6.4. Results Based on Mixed-Age Groups

Only three studies investigated differences between age groups within child and adolescent participants. Two found no differences [91,93], and another study [85] concluded that younger participants had a lower likelihood of sufficient PA.

The four investigations that included participants from age group 1 (children and adolescents) and 2 (adults) examined a total of 830 (600 female) participants with a mean age between 18 and 35+ years [102,103]. One used DB methods to gather PA [87], and the others used self-reported measurement methods. Two of the studies had good methodological quality and one study each had fair and poor quality. All four of them reported a significant reduction in PA. Steps [87], MVPA [102,103], and PA level [111] were considered. Hence, it can be concluded that studies that included participants from age groups 1 and 2 (in this context from 18 to 35+ years) have found reductions in PA using both DB and SR measurement methods.

Only Savage et al. [103] examined differences between gender. They revealed that MVPA was higher in males before the Covid-19 pandemic but not during the pandemic. They were also the only researchers to report age-related results, but these showed no difference between age groups.

Only two of the studies on adults addressed age-related differences. Ding et al. [79] first noted a sharp decline in steps following Covid-19 restrictions. Subsequently, the daily number of steps increased again. This increase was attenuated in those over 40 years of age compared to younger participants. Romero-Blanco et al. [100] detected significant differences in the increase of average minutes of PA in first- to third-year students.

The four studies with participants from age groups 2 and 3 (20 to over 65 years) included a total of 24,066 (11,561 female) participants with a mean age between 51.6 years [106] and 63.4 years [72].

Hino and Asami [66] andWang et al. [106] used DB measurement methods exclusively, and Bartlett et al. [72] and Ribeiro de Lima et al. [52] used SR measurement methods only. Bartlett et al. [72] were the only researchers who were able to identify a significant increase in total PA. In contrast, the results of Hino and Asami [66] and of Wang et al. [106] showed that daily/weekly steps significantly decreased. In addition, the prevalence of low daily steps increased significantly [106]. Furthermore, Ribeiro de Lima et al. [52] were able to show a significant reduction in domestic PA and free time PA but failed to detect a change in sports PA. The results indicate that DB measurement methods are more likely to detect a reduction in PA than SR methods.

Hino and Asami [66] and Wang et al. [106] were the only researchers who looked at both gender-related and age-related differences. The first concluded that steps decreased more in women than in men, and the second noted that the female gender was associated with a higher prevalence of low daily step count. Regarding age-related results, both drew similar conclusions, as steps decreased more in the younger than in the older participants [66] and older age was associated with a higher prevalence of frequent low daily steps [106], respectively.

A total of five studies included participants from all defined age groups. They included 72,315 (49,031 female) participants with an age range from 18 to 80 years. The quality assessment resulted in three good ratings, and one fair and one poor rating each. Di Sebastiano et al. [78] and McCarthy et al. [58] used DB measures, To et al. [56] used both DB and SR measurement methods and Obuchi et al. [94] and Woodruff et al. [108] used SR measures. All five studies revealed a significant decline in PA as operationalized by steps [56,78,94,108]; low PA and/or MVPA [78], and total PA [58] were examined for this purpose. Again, DB and SR measures revealed similar results.

Woodruff et al. [108] were the only researchers not to explore differences in PA between gender. Di Sebastiano et al. [78] and McCarthy et al. [58] both did not detect any differences between males and females. Obuchi et al. [94] revealed that changes in PA were smaller in men than in women and To et al. [56] postulated that men had a significantly larger step count than women, but these trends were consistent and indicated that the effects of the Covid-19 lockdown were not different for genders.

Three of these five studies also reported specific age-related results. Di Sebastiano et al. [78] did not observe any interactions between age and MVPA but stated that older adults (>55 years) recorded less low-intensity PA and steps than younger adults (25–55 years). The results of To et al. [56] pointed in the opposite direction, that older adults (>45 years) gained more daily steps than young adults (18–45 years). However, the results indicate that, in general, the effects of the Covid-19 pandemic did not have a differential impact on the age groups, apart from the fact that the negative effect of the pandemic was about twice as large among adults than Older Adults. The results of McCarthy et al. [58] were along the same lines and indicated that younger people engaged in more PA before the pandemic, but showed the least amount of PA within the pandemic situation. Hence, results suggested that older participants (≥65 years) showed a lower decrease in PA at the beginning of the Covid-19 pandemic, but a greater increase in PA as the pandemic situation continued.

### 3.7. Meta-Analysis

The overall model included a total of k = 139 r-to-z-transformed effect sizes out of 46 of the 57 studies, as 11 studies did not present necessary data within their publications and/or upon request. The Q-Test for heterogeneity revealed high significance (Q (df = 138) = 12,407.258, *p* < 0.001). The estimated average Fisher r-to-z transformed correlation coefficient based on the random-effects model revealed an overall low, negative, and-significant effect of the Covid-19 pandemic on PA (z = −0.18; 95% CI: −0.30–−0.06, *p* < 0.001).

As there were significant variances detected within and between studies regarding age (both *p* > 0.001), age was added as a moderator to the random-effect models. These models were fitted to the data for three multilevel meta-analyses matching the three different age groups (see above), though not revealing differences between the age groups regarding effect sizes.

The estimated average Fisher r-to-z transformed correlation coefficient based on the random-effects model was −0.08 (*p* > 0.05; 95% CI: −0.27–0.12, Q2(df = 33) = 5200.89, *p* < 0.001) for children and adolescents (k = 34), −0.17 (*p* < 0.05; 95% CI: −0.36–0.03, Q2(df = 72) = 4285.89, *p* < 0.001) for adults (k = 73) and −0.381 (*p* < 0.001; 95% CI: −0.65–−0.11, Q2(df = 31) = 2172.45, *p* < 0.0001) for older adults (k = 32). Therefore, PA changes due to the Covid-19 pandemic did not differ significantly from zero and the Q2 values indicate significant substantial to considerable heterogeneity of studies in all age-groups. The forest plots for the three age groups are displayed in Figure 3.

## 4. Discussion

This systematic review and meta-analysis aimed to examine the changes in PA patterns from before to during the Covid-19 pandemic and to provide an overview of global longitudinal changes in PA across all ages, with a further focus on gender and PA measurement method.

The results showed a heterogeneous picture, with evidence for both decreased and increased PA. However, the majority of the included studies (32/57) reported a significant decrease in PA and only 5 out of 57 showed a significant increase in the measured PA variables [57,72,84,100,109]. The remaining 14 studies showed both negative and positive changes in different domains of PA [52,55,64,65,70,73,76,77,79,81,83,93,104,110], and six studies did not indicate any change in PA [50,51,68,71,86,99]. In the following, the results are briefly summarized and discussed in the context of the existing literature.

### 4.1. Changes in PA according to Age

#### 4.1.1. Children and Adolescents

In total, 16 studies have been identified as investigating PA in children and adults. However, results are very mixed, as two studies found an increase [57,109], eight a decrease [53,63,69,75,80,85,91,105], four indicated mixed results [64,73,93,104] and two indicated no change in PA patterns [50,86]. Meta-analytical results revealed a slightly negative but non-significant trend towards decreases PA levels during the Covid-19 pandemic in children and adolescents.

Regarding studies that revealed an increase, there may be different reasons for this finding. First, the pandemic-related restrictions in some countries were not as severe as in other countries. In Sweden, for example, preschools were still open and, according to Guan et al. [112], children typically obtain their daily PA through active travel to school, physical education, and recess. In Germany, the preschools/kindergartens and schools were closed, but additional recreational time could be used for PA, which is reflected in an increase in this area in particular. These findings are new and do not coincide with previous studies which investigated PA patterns of young children before the Covid-19 pandemic. For example, Androutsos et al. [113], Dunton et al. [114], and Moore et al. [115] among others, showed a significant decrease in PA in this age group. Four studies produced mixed results and therefore found either increases and/or decreases of PA within their examined population.

The mixed results of Schmidt et al. [104] and Nigg et al. [93] on the one hand showed an increase, and a simultaneous decrease on the other. The increase was detected in total PA, nonorganized sports, and generally active days. As mentioned previously, one approach to explain this is that individuals in Germany had more time for PA due to the closure of schools, and the first lockdown was during the spring months in which the weather is favorable for outside activity. Furthermore, non-organized sports remained allowed if performed alone or with individuals from the same household. On the other hand, the decrease which was reported by both studies for organized PA in the context of sports is explained by the closure of sports clubs and gyms in Germany during the first lockdown [116]. Furthermore, Chen et al. [64] found a decrease in MVPA and no change in leisure-time PA, which is in complete contrast to the findings presented earlier. However, participants experienced no changes in their daily lives in Sweden, hence this decrease may not be due to Covid-19 related restrictions. Moreover, the investigation lasted for at least two years, therefore depicting normal changes depending on the maturation of participants (i.e., sport and exercise becomes less important, whereas peer groups and “hanging around” may have more impact in these age groups). Due to the long investigation period, seasonal influences may have influenced results here.

The majority of studies also revealed a decrease in PA in children and adolescents. Here it is important to notice that the study of Alonso-Martinez et al. [69], as the only study to use DB measurement methods was also the study with the youngest participants. As this study was conducted in Spain, high confinements were in place also for children, possibly accounting for the decrease in PA found in this age group. The remaining studies were conducted in different countries making results due to differences in confinement strategies difficult to compare. Two studies found no change in PA [50,86].

Regarding measurement outcomes, these studies used several outcomes, which, however, may not depict the complex nature of child and adolescent PA, which is composed of many different aspects including leisure-time PA, LPA, MPA, VPA, or sport and exercise. Hence, special outcomes that have been defined in the different studies may not depict PA in its complete nature. Moreover, most studies used SR measures, which are known to be susceptible to over- or underreporting, especially in child populations [61,117,118,119,120]. Moreover, it might be that SR measures were not self-administered in this age group, as legal guardians probably answered the questions.

In general, these outcomes fit with the findings of the current literature, not included in the present analyses (e.g., due to a cross-sectional design), which also states that adolescent PA decreased significantly during the period of the Covid-19 pandemic [10,115]. However, based on meta-analytical results, it needs to be mentioned that the change in PA due to the pandemic seems to be slightly negative in children and adolescents, but not significantly. Moreover, very young children aged below 10 years were only the subject of five studies and are therefore underrepresented in the current investigation.

#### 4.1.2. Adults

A total of 21 studies investigated PA patterns in adults. Results were also mixed, as two studies found an increase in PA [84,100], 10 found a decrease, six revealed mixed results [70,76,77,79,83,110] and three did not detect any change due to Covid-19 restrictions [51,68,71]. Meta-analytic results also revealed a slight negative, but non-significant trend towards a decrease in PA due to the Covid-19 pandemic.

Regarding the results of increased PA, it is striking that these two research groups both examined participants from Spain and from a similar setting and environment, one being health science students and the other nursing students. It is difficult to imagine that this is the reason for the contrasting results because, after all, Martinez-de-Quel et al. [90], Sanudo et al. [101], and Savage et al. [103] also examined young students. Due to the special and especially health-oriented field of study, it might be that these students pay special attention to health and exercise. Similarly, Mandic et al. [121] observed that most of the medical students investigated were aware of the benefits of PA and were regularly active. In general, however, a reduction in PA among students is evident, not only in this review but also in the current literature [47,122,123]. The results seem counterintuitive at first glance, regarding the restrictions imposed by the government. These were very strict in Spain (as compared to, for example, Sweden; [67]), with the population not allowed to leave their homes without valid reason and outdoor exercise or sports prohibited (see corresponding studies). Since other recent studies [123,124] also showed a decrease in PA in this age group, the results suggest that PA (with exceptions) was significantly decreased in individuals between 20 and 35 years of age.

Regarding studies that revealed mixed results, only the domains time walking and engaging in MPA during leisure time [76], cycling, and total MPA showed an increase; all other domains revealed a (significant) decrease in PA (e.g., PA when commuting, team sports). Looking at the restrictions, especially in France (the population was allowed to go out for a maximum of 1 h per day and had to stay within 1 km of their house), these results stand to reason as especially commuting activities and organized sports activities were no longer necessary. Here, only the results of Cheval et al. were counterintuitive, as they found an increase in walking and MPA [76]. Here, participants probably compensated for hard lockdown restrictions by walking around the block or increased engagement in-home workouts.

Decreases in PA were observed in ten studies conducted in multiple countries such as Italy, the United States, China, Japan, Spain, Singapore, and South Korea, which, however, had comparable Covid-19 related restrictions during their measurement periods, including social distancing measures and prohibition of large gatherings (including sports facilities). Therefore, the decrease in PA may mainly arise from (1) stay-at-home and work-from-home orders and (2) a ban on organized sports groups. Measurement outcomes and methods were also mixed within these studies. Decreases were found regarding step count, MPA, MVPA, total PA, VPA, or MET minutes/week, not allowing for the generalizing of decreases to specific outcome measures. SR and DB-based measurements were also represented in these studies (six SR, three DB, and one study using both). Therefore, results may be attributed to the overall repercussions of the Covid-19 pandemic. The decrease in PA in this age group is in line with other Covid-19 related literature not included in the present investigation, such as recent literature by Górnicka et al. [125] and Bourdas and Zacharakis [126]. In addition, three studies did not reveal any changes in PA levels before and during the Covid-19 pandemic [51,68,71].

Taken together, meta-analytical results also support the assumption of a decrease in PA in adults, although not significant. However, it needs to be taken into account that the results for adults aged between 45 and 65 years of age are completely missing so far. Hence, results cannot be generalized to the general adult population. Here, further investigations with middle-aged adults are warranted.

#### 4.1.3. Older Adults

For over 60-year-olds, a total of seven studies was identified. However, no reliable conclusion can be drawn based on the studies included in this review, as three studies showed a significant decrease [92,95,98], three showed mixed results [55,65,81], and one study did not reveal any change in PA patterns [99]. Elderly people, in particular, are at increased risk of severe Covid-19 disease progression [127], so special care should be taken in this age group to comply with applicable government regulations to protect themselves first and foremost. In contrast to children, adolescents, and (young) adults, older adults seem to significantly decrease their PA levels during the Covid-19 pandemic, based on meta-analytical results.

Interestingly, no study revealed a conclusive increase in PA during the Covid-19 pandemic in older adults. Given the restrictions (UK, Scotland, Japan, United States, Spain), it is surprising that there were older adults who were able to increase their PA in the studies that revealed mixed results, despite the circumstances. In the study of Esain et al. [81], for example, it is surprising that total PA, walking, and cleaning have decreased, whereas exercising and dancing significantly increased. Hence, outdoor activities may have been replaced to compensate for older adults’ disappointment by low-social contact sports. This finding is also supported by Suzuki et al. [65], who revealed light, moderate, and heavy housework increased significantly during the Covid-19 pandemic, but only in highly active participants. These mixed effects may result from the circumstance that access to PA and exercise is hardly limited for older adults as they experience more barriers with access to digital alternatives and also have a lack of access to technology or know-how [128,129,130]. However, there is clear evidence in the literature that PA also reduced in this age group [131,132,133].

This assumption is underlined by the three studies that revealed clear decreases in PA. Results need to be interpreted with caution, as SR measures, as well as DB measures, were used, with SR measures only using a single item question [95] or a very brief questionnaire [98]. Altogether, it is conspicuous that only leisure-time PA outcomes were used here (e.g., total PA, MPA, LPA, walking, living-activity, steps). Hence, organized sports did not seem to play any role in the decrease of PA of older adults. However, leisure-time PA also decreased as measured by the above-mentioned outcomes, showing impacts of Covid-19 related restrictions (i.e., social distancing, lockdown, stay-at-home orders) or fear of going outside and of probable contraction of the Sars-Cov-2 virus.

#### 4.1.4. Larger Age Ranges

The remaining 13 studies investigated participants from multiple age groups. Four studies investigated age groups 1 and 2 [87,102,103,111], four groups 2 and 3 [52,66,72,106] and five studies investigated participants of all ages [56,58,78,94,108]. Out of these, only one study revealed an increase in PA [72]. This is surprising, as it investigated participants aged over 50. This age group (60 and above) especially has been shown earlier to decrease their PA, and this, therefore, is contrary to expected results. Only one study produced mixed [52] results in participants between 50 and 70 years of age, finding no increase, but some decrease or no change depending on PA outcome of choice. The authors showed decreases in domestic PA and free time PA, but no significant change in sports PA and total PA, which is contrary to the findings of Esain et al. [81] discussed above. However, results need to be interpreted with caution as only 34 physically inactive women were included in the study by Ribeiro de Lima et al. [52]. Whereas one study revealed mixed results, the remaining 11 studies pointed decisively to decreased PA irrespective of age group, outcomes (i.e., steps, MVPA, PA levels), and measurement method (SR and DB) investigated, commonly confirming the results presented above.

Six of the studies also explicitly reported age-related results. Two did not find any difference in PA due to the Covid-19 pandemic [78,103], one revealed that older participants (over 60 years) walked fewer steps per day [106], and three studies showed the exact opposite, that older participants (at least 45 years old) walked more steps than younger participants [56,58,66]. Here, multiple aspects may play a role in interpreting these contrary results. The impact of country-related results may play a role, as Wang et al. [106] investigated Chinese participants, whereas the other three conducted their studies in the UK, Australia, and Japan, where different measures may have been applied. Moreover, time points of investigations might have differed regarding Covid-19 restrictions. Different demographic measures and sample characteristics may also play a role. Interactions between before- and within-Covid-19 PA [58] may point to the possibility that younger people were more affected by the closure of sports facilities and organized sports than older people.

### 4.2. Change in PA according to Measurement Methods

Most researchers used SR measurement methods in a total of 40 studies to detect differences in PA, whereas 11 studies used DB measures and six studies used both SR, and DB measurements. In four of the 40 studies using SR (including those that used both SR and DB), a significant increase in PA was disclosed in at least one of the activity patterns examined [72,84,100,109]. 11 studies produced mixed results when using SR methods [52,64,65,70,73,76,81,83,93,104,110], and 19 revealed a decrease. Six studies found no change [50,51,68,71,86,99].

Only 11 studies used DB measurements. Of these, none revealed an increase, one produced mixed results [55] and 10 found a decrease [58,66,69,78,87,88,92,96,97,106]. No study revealed the absence of any change.

Of the six studies using both, SR and DB methods, Nyström et al. [57] found increases in PA independent of the measurement method. Two studies produced mixed results [77,79]. Here, Curtis et al. [77] found significant decreases in LPA but no change in MVPA when measured with DB methods. In terms of recreational PA measured via SR, decreases, increases, and no changes were found. Ding et al. [79] used SR measures for before-Covid-19 assessment and used step count as a DB measure, which they found to have decreased, but then increased again during the Covid-19 pandemic. The remaining three studies using mixed-method approaches revealed a decrease in PA [56,63,101].

Comparing the findings of the studies which used SR methods with those of the studies which used DB methods, one can detect a difference. Mishra et al. [55] failed to detect a significant reduction in LPA and MVPA, but all other domains examined by these 11 research groups produced significant decreases in PA. In addition to step measurements, other levels of PA such as intensity (MPA, VPA, MVPA) or duration (weekly minutes of tracked PA) were also recorded.

### 4.3. Change in PA in Males and Females

A total of 23 studies reported participants’ gender-specific PA, 13 of them finding a decrease in both genders. Cheval et al. [76] showed a decrease in two measured areas and an increase in two others for both genders. Both Nigg et al. [93] and Schmidt et al. [104] revealed an increase in PA in two domains and a decrease in one domain in girls and boys. In addition, Franco’s research group [83] identified an increase in two domains and no change in two other areas, also for both genders. Richardson et al. [99] found no changes in the areas of PA studied for either gender. Since there were no gender-specific restrictions, it is comprehensible that the constraints affected both genders equally.

Baceviciene et al. [70], Elnaggar et al. [80], and Sekulic et al. [105] all found a reduction in PA for boys/men only, while Chen et al. [64] observed a reduction in PA only for girls. In the countries of the first three studies, there were very strict measures taken by the government to contain the virus, including imposing curfews. Since boys and men, respectively, tend to have higher PA levels than girls or women [25,26,27], they may have been more affected by these restrictions than their female study counterparts. Chen et al. [64] examined 15-year-old adolescents in Sweden, who may have been affected by school closures or home schooling (there were no restrictions for pupils below the 9th grade), and the boys may have been more likely to use free time to exercise or play sports. In contrast, one investigation [100] noted a significant increase in PA only for women. Since Spain had very strict regulations and people were only allowed to leave the house for necessary purposes, these results seem rather contradictory.

Regarding differences between both genders, nine of the 23 studies reported that activity patterns did not differ significantly, and another seven studies did not report differences between genders at all. The research groups headed by Gilic et al. [85] and Sekulic et al. [105] revealed significantly higher PA for boys compared to girls. Similar findings were revealed by Obuchi et al. [94], To et al. [56], Hino et al. [66], and Wang et al. [106], who also found higher PA levels in the respective domains for men versus women. The fact that men had higher levels in the individual fields of PA is in line with typical studies and has already been examined frequently [25,26,27].

On the other hand, the study by Franco et al. [83] showed that the number of men reaching recommended PA levels decreased, while more women reached recommended levels during the pandemic than before. The research by Van Uffelen et al. [134] in a similar population provides a possible explanation. According to this, women, in contrast to men, perceive the improvement of external appearance as a motivating factor for PA. In times of a nationwide lockdown, there is sufficient opportunity to “improve” this external appearance.

In summary, PA decreased significantly in both genders in most cases, and higher values for PA were more likely to be found in males than in females. This opinion is supported by the studies of García-Tascón et al. [135], Maugeri et al. [19], and Rodriguez-Larrad et al. [136], who also revealed a reduction in PA in both genders. The former two were able to detect a difference between the genders, with higher PA values for men, while the latter registered no difference.

### 4.4. Additional Influencing Factors on PA during the Covid-19 Pandemic

Another factor influencing PA levels during the Covid-19 pandemic might be the place of residence. In rural areas, it is often easier to find suitable areas for sports activity, even within a limited radius, and usually rural houses and apartments also offer more space to practice PA. This conjecture is confirmed by Beck et al. [137]. A comparison between people from urban and rural areas revealed a positive impact of living in rural areas [138]. Thus, PA either increased significantly or at least did not show a significant decrease compared to that of urban dwellers [53]. Rice et al. [139] came to a similar conclusion. However, they examined the change in PA only indirectly by analyzing recreational behavior. Residents of urban areas reduced their activities significantly more than residents of rural areas. Older (pre-Covid-19) investigations tend to favor urban areas when it comes to higher PA [140], but recent studies parallel to this review also show higher PA levels in rural areas [141,142]. Nigg et al. [93] showed that children and adolescents residing in densely populated areas showed less favorable PA changes than children and adolescents living in sparsely populated areas.

Furthermore, socio-demographic, or geo-social differences may play a role. Within Canada, Quebec recorded less MVPA, LPA, and steps compared to other regions [78], but in general PA decreased significantly in all regions. Since there is no more detailed information on the participants and restrictions, the reasons for this observation can only be speculated on. Likewise, Rhodes et al. [143] demonstrated a significant decline in MVPA in a similar sample of Canadians, but they did not investigate regional differences.

To limit the spread of the virus and minimize its negative impact, each country developed its own strategy to fight the virus leading to international differences in Covid-19 restrictions of confinements. Therefore, regarding the distribution of countries (and therefore the weight of country-specific Covid-19 regulations within the meta-analytic results), most of the included studies have been conducted in Spain and examined the change in PA of Spanish residents. The five Spanish research groups provide contradictory findings, as three found a significant decrease in PA [90,91,101], while the other two found divergent results, namely a significant increase in PA [84,100]. The reasons for the different results of these two studies have already been discussed in detail above. The very harsh restrictions imposed by the Spanish government and the evidence from numerous other studies [8,144,145,146,147] tends to suggest that PA has significantly decreased in Spanish participants. Four studies provided insight into the change in PA during confinement in the United Kingdom. While three studies found a significant decrease in measured PA domains [58,95,103], the findings of Richardson et al. [99] suggest that PA increased during this period. However, the evidence was negligible. This is also reflected in other studies that were not included in the review. Faulkner et al. [148] and Ruiz et al. [149] each showed a decrease in PA for Britons, whereas Spence et al. [150] indicated that most of the sample maintained or even increased their PA. However, the percentage of participants meeting the PA guidelines was significantly lower than in previous years. One possible explanation for the sporadic increases displayed in PA could have been the exceedingly pleasant and sunny weather in May 2020 [151]. For example, although gyms were closed, individuals were allowed to leave the house or apartment once a day to exercise [103]. Therefore, the significant declines in PA shown are somewhat surprising, as the restrictions were rather moderate compared to countries such as Spain or China. The results of the review suggest that PA significantly reduced during the lockdown in both China [54,88,106] and Croatia [53,105]. Looking at the results from China, one possible explanation for the decline in PA is the stringent mitigation policies. For example, He et al. [88] reported that only one person from each household was allowed to go out every two to three days to buy essentials. A similar strict approach was used by the Croatian government. Sekulic et al. [105], for instance, stated that sports and fitness centers and public parks were closed, but there was no strict ban on various forms of individual training. According to Cheval et al. [76], the imposed restrictions had a larger effect on the field of PA in transportation (commuting) for Swiss than for French participants. Overall, the time spent commuting decreased in both countries, but it is surprising that the effect was larger in Swiss participants, since the restrictions in this country were much looser than in France. To the best of the author’s knowledge, this comparison is the first of its kind. Examinations regarding PA levels during the Covid-19 pandemic concerning the country of examination and its respective restrictions during the pandemic are previously missing. The lower negative impact of the pandemic can also be explained in terms of containment measures (Ireland: staying at home as much as possible, no more than four people meeting outdoors, 5-km travel limit; Croatia: extensive social distancing measures, sports and fitness centers closed).

### 4.5. Summary of Discussion and Comparison

In general, it is important to point out that there is always a risk of recall bias in SR methods. Numerous studies have suggested that this can lead to both underestimation [61,118,152] and overestimation [119,120] of one’s own PA. In this respect, one must assume that the findings of DB measurements are more robust and therefore come closer to the true extent of PA. However, differences in minimal wear-time, epoch length, and/or devices also make DB data difficult to compare [153].

In summary, the results of the current review and meta-analysis are consistent with those of the three previous reviews conducted by Stockwell et al. [22], López-Valenciano et al. [23], and Rossi et al. [24]. Here, the importance of age and gender as important determinants was also highlighted [22,23]. All reviews predominantly identified studies that reported a significant decline in PA. In the case of López-Valenciano et al. [23], nine of ten papers did, but the authors rated the quality of evidence as low. Unfortunately, the results of Stockwell et al. [22] do not provide an exact number of studies with a negative effect on PA. “The (...) review of 66 studies demonstrated that the majority of studies found that PA declined (...) during the COVID-19 pandemic lockdown, regardless of the subpopulation or the methodology used.” [22]. Rossi et al. [24] found a decrease in 57 out of 84 studies, whereas only four found an increase and 14 reported either no change or no results. In the current analysis, five out of 57 studies found an increase, 32 a decrease, 14 revealed mixed results and six reported no change.

However, one of the reasons for the similar findings may be that the included studies overlap. In total, the present review included 31 studies from the other three reviews (nine included in Stockwell et al., three included in López-Valenciano et al., and nine included in Rossi et al.) on this topic. However, it must be mentioned that, despite different underlying inclusion criteria, all three reviews achieved similar findings.

### 4.6. Strengths and Limitations

The current investigation has several major strengths. First, it is, to the best of the authors’ knowledge, the first only to include longitudinal research designs that had at least one measurement before the Covid-19 pandemic and at least one measurement within the Covid-19 pandemic, making results interpretable in terms of causality and relatedness and accounting for within-person changes of PA. More advantages include the capability to identify and associate events with specific exposures and to further specify these exposures in terms of presence, timing, and chronicity, the tracing of changes over time in specific individuals within the cohort, using prospective data collection to eliminate recall bias among participants, and the ability to correct for the cohort effect by accounting for the influence of components on an individual basis [154,155]. Furthermore, this investigation acknowledged the point made by other reviews by including age and gender as an important determinant of PA, especially during the pandemic [22,23,24], and exploratively investigated the difference of results obtained by SR or DB measurement of PA. Last, it is the first study to date to use a meta-analytic approach to quantify the effects of PA changes due to the Covid-19 pandemic.

However, this systematic review and meta-analysis also has some limitations to be considered. Looking at the evidence included in the review, it must be noted that, except for three investigations from Saudi Arabia [51,80,111], no suitable studies on the African continent were found. Therefore, no conclusions about PA during the Covid-19 pandemic in Africa can be drawn. Furthermore, very few of the included studies reported either an a priori or a posteriori power analysis to obtain an estimate for adequate sample size and power estimation. Some of the studies showed a very small sample size [52,55,77,80,81,92,101]. However, the sample size did not appear to be too small in the remainder of the included studies. A shortcoming of a large sample size was that the studies in question could not be included in the synthesis by age group because, apart from Schmidt et al. [104], these authors did not report age-specific results. Moreover, in each synthesis, only one variable was subsumed, while the others were left out. This means, for example, that the change in PA of women in Spain between 20 and 35 years of age measured with objective measurement methods was not examined, but only that of women, or inhabitants of Spain, or 20- to 35-year-olds, or results of objective measurement methods. Hence, no study incorporated all variables of interest. An additional limiting factor is that many of the included studies used questionnaires that do not fully meet the generally accepted scientific standards for good reliability and, above all, validity [156,157]. The review includes a total of seven studies [72,74,75,82,84,95] that did not provide information on the quality criteria of their measurement methods. More than 30 of the included studies did not reach the quality threshold for validity, showing only low or moderate validity. Hence, results of studies using SR methods need to be interpreted with caution, and reliability and validity issues should be taken into account when interpreting their results.

Although the criteria required the inclusion of healthy participants only, cardiovascular, or musculoskeletal diseases were frequently present, especially in elderly subjects (see [65]), which may have influenced PA, especially in the older population. However, since these are normal age-related concomitants, they were not considered further. Another limitation to be considered is that the influence and the severity of lockdown or restrictions and further influencing factors, e.g., sociodemographic factors, on activity behavior were not investigated. In addition, the language was limited to English and German.

## 5. Conclusions and Implications

During the Covid-19 pandemic, PA decreased significantly in all age groups, in males, and females, and in most countries. The findings of Martínez-de-Quel et al. [90], for example, suggest that the negative effect of insufficient PA is greater on previously active individuals. Moreover, during the pandemic, negative effects on mental health due to increased inactivity were found [158]. Since the positive effects of PA are also well documented, governments should focus on allowing and enabling PA and exercise under current safety and hygiene regulations. The position paper of the Association for Aerosol Research [159] assumes, for example, that almost no infections from aerosol particles occur during non-contact sports activities outdoors. That active exercise during a pandemic enhances both, physical and mental health, is, for example, supported by the study of Wilson et al. [160]. While the control group experienced a decrease (albeit not significant) in PA variables, the intervention group significantly improved these values as well as sleep quality and quantity, fruit and vegetable intake, and mental health. Another way to take advantage of sports offerings in times of restrictions and, in some cases, curfews is to move these offerings into the digital space. Parker et al. [161] found that users of digital platforms were more likely to adhere to PA guidelines than nonusers during the pandemic. In addition, there are numerous recommendations on how to have a decent workout even at home [162,163,164,165].

Future research should focus primarily on collecting data on the same forms of PA with the same measurement instruments. This will increase the comparability of results across studies. The experience of this review shows that steps, intensity (MPA, VPA, or MVPA), and total PA in time (hours/MET) are suitable variables. For the reasons stated above, it is recommended that objective methods be used, as these are most likely to collect actual PA. Regarding the study design, it is recommended to use a longitudinal design. A finding of this review is that studies with a very broad sample necessarily require subgroup analysis (e.g., age, gender, or regional differences), because the results of the overall sample are of limited value and generalizability (see [58,78,106]). 

Although attempts have been made to examine the impact of the respective lockdown measures on PA, there is yet no consistent measure to compare these policies. Stockwell et al. (2021) therefore called for the development of a “lockdown scale”, which should be addressed in future research.

## Figures and Tables

**Table 1:** PICOS criteria for inclusion and exclusion.

	Inclusion	Exclusion
Population	Healthy human subjects: no restriction on age, demographic variables, or geographical region	Groups of special interest not representing the general population (e.g., professional athletes) as well as studies in specialized settings (e.g., hospitals)
Intervention	Quasi-experimental: during the Covid-19 pandemic	If (1) only a single measurement was taken (cross-sectional) or if (2) multiple measurements were taken, but conducting retrospective assessments (i.e., asking within the pandemic questions about before the pandemic)
Comparison	Change in PA from before- to within-Covid-19 pandemic	
Outcome	Any form of PA, either subjectively (self-reports) or objectively (i.e., accelerometry) measured	Studies investigating other health-related behaviors and not reporting PA. For the meta-analysis, studies not providing information for effect size estimation were also excluded.
Study type	Only longitudinal studies with at least one measurement before the Covid-19 pandemic, as well as at least one measurement within the Covid-19 pandemic, were included: Observational studies, cohort studies, and pre-post tests were included.	Literature reviews, abstracts and conference proceedings, study protocols, editorials or commentaries, and letters to the editors not including any original data were excluded.

**Table 3:** Quality Assessment and Risk of Bias within included studies.

Author(s) (Year)	1	2	3	4	5	6	7	8	9	10	11	12	Quality Rating
Aegerter et al. (2021) [68]	Y	Y	Y	Y	Y	Y	Y	NA	N	Y	Y	NA	Good
Al-Musharaf et al. (2021) [51]	Y	Y	Y	Y	Y	Y	Y	NA	N	Y	N	NA	Good
Alonso-Martinez et al. (2021) [69]	Y	Y	Y	Y	CD	Y	Y	NA	N	Y	N	NA	Fair
Baceviciene and Jankauskiene (2021) [70]	Y	Y	Y	N	CD	Y	Y	NA	N	Y	N	NA	Poor
Barone Gibbset al. (2021) [71]	Y	Y	Y	Y	CD	Y	Y	NA	N	Y	N	NA	Fair
Bartlett et al. (2021) [72]	Y	Y	Y	Y	CD	Y	Y	NA	N	Y	Y	NA	Good
Bronikowska et al. (2021) [73]	Y	Y	Y	Y	Y	Y	Y	NA	N	Y	N	NA	Good
Buoite Stella et al. (2021) [74]	Y	Y	Y	Y	CD	Y	N	Y	Y	Y	Y	NA	Good
Chaffee et al. (2021) [75]	Y	Y	Y	Y	Y	Y	Y	NA	N	Y	Y	NA	Good
Chen et al. (2021) [64]	Y	Y	Y	Y	CD	Y	N	NA	N	Y	Y	NA	Poor
Cheval et al. (2020) [76]	Y	Y	Y	Y	Y	Y	Y	NA	N	Y	N	NA	Good
Curtis et al. (2021) [77]	Y	Y	Y	Y	CD	Y	Y	NA	N	Y	Y	NA	Good
Di Sebastiano et al. (2021) [78]	Y	Y	Y	Y	CD	Y	Y	NA	N	Y	Y	NA	Good
Ding et al. (2021) [79]	Y	Y	Y	Y	CD	Y	Y	NA	N	Y	Y	NA	Good
Elnaggar et al. (2020) [80]	Y	N	Y	CD	CD	N	Y	NA	NR	Y	Y	NA	Poor
Esain et al. (2021) [81]	Y	Y	Y	Y	CD	Y	Y	NA	N	Y	Y	NA	Good
Folk et al. (2021) [82]	Y	Y	Y	Y	CD	Y	Y	NA	N	Y	Y	NA	Good
Franco et al. (2021) [83]	Y	Y	Y	Y	CD	Y	Y	NA	N	Y	N	NA	Fair
Gallego-Gomez et al. (2020) [84]	Y	Y	Y	Y	CD	Y	N	NA	Y	Y	Y	NA	Fair
Gilic et al. (2020) [85]	Y	Y	Y	Y	CD	Y	Y	NA	Y	Y	N	NA	Good
Gilic et al. (2021) [86]	Y	Y	Y	Y	CD	Y	Y	NA	Y	Y	N	NA	Good
Giuntella et al. (2021) [87]	N	Y	N	Y	CD	Y	Y	NA	NR	Y	Y	NA	Poor
He et al. (2020) [88]	Y	Y	Y	Y	CD	Y	N	NA	Y	Y	Y	NA	Good
Hino et al. (2021) [66]	Y	Y	Y	N	N	Y	Y	NA	Y	N	Y	NA	Fair
Koohsari et al. (2021a) [89]	Y	Y	Y	N	CD	Y	Y	NA	N	Y	N	NA	Poor
Martinez-de-Quel et al. (2020) [90]	Y	Y	Y	Y	CD	Y	Y	NA	N	Y	N	NA	Fair
McCarthy et al. (2021) [58]	Y	Y	Y	Y	CD	Y	Y	NA	NA	Y	Y	NA	Good
Medrano et al. (2020) [91]	Y	Y	Y	Y	Y	Y	Y	NA	N	Y	N	NA	Good
Mishra et al. (2021) [55]	Y	Y	Y	N	CD	Y	Y	NA	Y	Y	N	NA	Fair
Miyahara et al. (2021) [92]	Y	N	N	N	CD	Y	Y	NA	N	N	N	NA	Poor
Munasinghe et al. (2020) [63]	Y	Y	Y	Y	CD	Y	N	NA	N	Y	Y	NA	Fair
Nigg et al. (2021) [93]	Y	Y	N	Y	Y	Y	Y	NA	Y	Y	Y	NA	Good
Nyström et al. (2020) [57]	Y	Y	Y	Y	CD	Y	N	NA	Y	Y	Y	NA	Fair
Obuchi et al. (2021) [94]	Y	Y	Y	N	CD	Y	Y	NA	Y	Y	Y	NA	Good
Okely et al. (2021) [50]	Y	Y	Y	N	Y	Y	N	NA	NR	Y	N	NA	Poor
Okely et al. (2020) [95]	Y	Y	Y	Y	CD	Y	N	NA	N	Y	N	NA	Poor
Ong et al. (2020) [96]	Y	Y	Y	Y	CD	Y	Y	NA	Y	Y	Y	NA	Good
Park et al. (2021) [97]	Y	Y	N	Y	CD	Y	Y	NA	N	Y	N	NA	Poor
Perez et al. (2021) [98]	Y	Y	Y	N	CD	Y	Y	NA	Y	Y	N	NA	Good
Riberiro de Lima et al. (2021) [52]	Y	Y	Y	CD	CD	Y	Y	NA	NR	Y	N	NA	Fair
Richardson et al. (2020) [99]	Y	Y	Y	Y	CD	Y	Y	NA	Y	Y	Y	NA	Good
Romero-Blanco et al. (2020) [100]	Y	Y	Y	Y	Y	Y	Y	NA	Y	Y	N	NA	Good
Sanudo et al. (2020) [101]	Y	Y	Y	Y	CD	Y	Y	NA	N	Y	Y	NA	Fair
Savage et al. (2021) [102]	Y	Y	Y	N	CD	Y	Y	NA	N	Y	N	NA	Poor
Savage et al. (2020) [103]	Y	Y	Y	Y	CD	Y	Y	NA	N	Y	Y	NA	Good
Schmidt et al. (2020) [104]	Y	Y	Y	Y	CD	Y	Y	NA	N	Y	N	NA	Good
Sekulic et al. (2020) [105]	Y	Y	Y	NR	CD	Y	Y	NA	NR	Y	N	NA	Poor
Suzuki et al. (2020) [65]	Y	Y	Y	Y	CD	Y	N	NA	Y	Y	N	NA	Fair
To et al. (2021) [56]	Y	Y	Y	Y	CD	Y	N	NA	Y	Y	Y	NA	Poor
Wang et al. (2020) [106]	Y	Y	Y	Y	CD	Y	Y	NA	Y	Y	Y	NA	Good
Wilson et al. (2021) [107]	Y	Y	Y	CD	CD	Y	Y	NA	NR	Y	N	NA	Poor
Woodruff et al. (2021) [108]	Y	Y	Y	CD	CD	Y	N	NA	N	Y	Y	NA	Poor
Wunsch et al. (2021) [109]	Y	Y	Y	N	CD	Y	Y	NA	N	Y	N	NA	Poor
Yang and Koenigstorfer (2020) [110]	Y	Y	Y	Y	Y	Y	Y	NA	N	Y	N	NA	Good
Zenic et al. (2020) [53]	Y	Y	Y	NR	CD	Y	Y	NA	NR	Y	N	NA	Poor
Zheng et al. (2020) [54]	Y	Y	N	Y	CD	Y	Y	NA	Y	Y	N	NA	Fair

Note: Y, yes; N, no; CD, cannot determine; NA, not applicable; NR, not reported. (1) Objective clearly stated; (2) eligibility criteria described; (3) representative patient population; (4) all eligible participants enrolled in the study; (5) sample size sufficient; (6) invention description; (7) outcome measures specified; (8) outcome assessor-blinded; (9) loss to follow-up; (10) statistical analysis of outcome measures before and after the intervention; (11) interrupted time series design; (12) individual data used for group-level effects.

## Data Availability

Data can be obtained from the corresponding author upon request.

## Supplementary Materials

The following are available online at https://www.mdpi.com/article/10.3390/ijerph19042250/s1, Table S1: Individual search terms for databases.
