# Supplementary material for: Changes in Physical Activity Patterns Due to the Covid-19 Pandemic: A Systematic Review and Meta-Analysis
Source: Int J Environ Res Public Health. 2022 Feb 16;19(4):2250. doi: 10.3390/ijerph19042250 (PMC8871718; doi:10.3390/ijerph19042250)
Supplement: Supplementary file 1 [file ijerph-19-02250-s001.zip › ijerph-1541260-supplementary.pdf]

Supplementary Table S1.

Supplementary Table 1. Individual search terms for databases (10.06.2021).

Database	Searchterm	Matches	Added to project (without doublettes)
**Web of Science**	(TS=(COVID-19 OR SARS-CoV-2 OR COVID-19 pandemic OR Corona virus) AND TS=(Physical Activit* OR Physical Exercise* OR Home Workout) AND PY=(2020 OR 2021)) *AND* **LANGUAGE:** (English OR German)	1545	1545
**Scopus**	(TITLE-ABS-KEY(COVID-19) OR TITLE-ABS-KEY(SARS-CoV-2) OR TITLE-ABS-KEY(COVID 19 pandemic) OR TITLE-ABS-KEY(Corona virus) AND TITLE-ABS-KEY(Physical Activit*) OR TITLE-ABS-KEY(Physical Exercise*) OR TITLE-ABS-KEY(Home Workout)) AND (LIMIT-TO (PUBYEAR, 2020) OR LIMIT-TO (PUBYEAR, 2021)) AND (LIMIT-TO (LANGUAGE,"English") OR LIMIT-TO (LANGUAGE,"German"))	2801	1489
**Pubmed**	((Covid-19[Title/Abstract]) OR (Coronavirus[Title/Abstract]) OR (SARS-CoV-2[Title/Abstract]) OR (Covid 19 pandemic[Title/Abstract])) AND ((Physical Activit*[Title/Abstract]) OR (Physical Exercise*[Title/Abstract]) OR (Home Workout[Title/Abstract])) AND ((english[Filter] OR german[Filter]) AND (2020:2021[pdat]))	1275	148
	**Total:**	**5621**	**3182**
